# Lotus fiber-derived scaffolds for enhanced cultured meat production: Quality and sustainability

**DOI:** 10.1016/j.bioactmat.2025.06.048

**Published:** 2025-06-27

**Authors:** Yueren Wu, Yajun Li, Qing Yang, Chaoyong He, Jianping Tang, Liyang Shi, Jianwu Dai, Can Zhang

**Affiliations:** a Hunan Research Center of the Basic Discipline for Cell Signaling, College of Biology, Hunan University, Changsha, 410000, China; b Chenxi Xinchuang Biological Technology Co., Ltd, Zhenjiang, 21200, China; c State Key Laboratory of Molecular, Developmental Biology, Institute of Genetics and Developmental Biology, Chinese Academy of Sciences, Beijing, 100101, China

**Keywords:** Cell-cultured meat, Lotus fiber scaffold, Porcine muscle stem cells, Myogenesis, Multi-omics analysis

## Abstract

The expanding global population intensifies demand for sustainable protein sources. Cell-cultured meat (CM) offers a promising alternative to conventional meat production but faces challenges in scalability and food-grade scaffold design. Current scaffolds often fail to replicate muscle tissue's structural and mechanical properties or support large-scale CM production. Moreover, the sensory and nutritional qualities of CM remain understudied. Here, we developed a novel lotus fiber-based natural plant fiber (NPF) scaffold mimicking native muscle tissue architecture. Porcine muscle stem cells (pMuSCs) were cultured on the NPF scaffold (pMuSCs-NPF), and their viability, proliferation, and differentiation were evaluated. The NPF scaffolds exhibited high biocompatibility and promoted pMuSCs alignment and differentiation into organized myotubes, as evidenced by enhanced expression of myogenic markers (MYOD, MYOG, MyHC) and extracellular matrix (ECM) components (desmin, fibronectin). Multi-omics analyses revealed substantial upregulation of genes and proteins associated with muscle development and ECM remodeling in pMuSCs-NPF compared to conventional plastic culture. Sensory and nutritional analyses indicated that the resulting CM closely resembled traditional meat in appearance, texture, and nutritional profile, with comparable levels of protein and essential amino acids. Moreover, the NPF scaffold demonstrated scalability and supported adipogenic differentiation, which is vital for imparting meat-like flavor and texture. These findings establish NPF scaffolds as a viable and cost-effective platform for sustainable CM cultivation.

## Highlights

• Lotus fiber: Sustainable, edible, animal-free, and scalable for cultured meat production.
• NPF scaffold promotes the differentiation and maturation of pMuSCs into myotubes.
• The resulting cell-cultured meat (CM) matches traditional meat in appearance, organoleptic characteristics, and nutrition.
• Pork belly prototype with both muscle and fat tissues shows whole-cut CM potential.

## Graphical abstract

**Figure:**
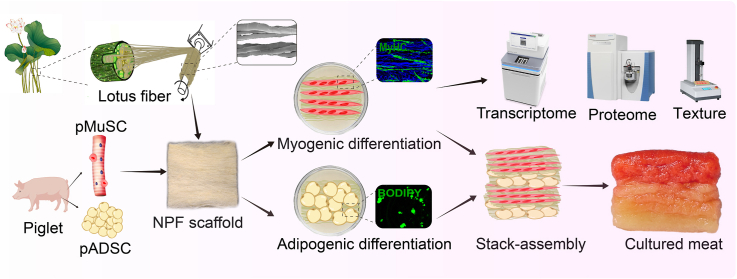

## 1 Introduction

Meat serves as a concentrated source of essential nutrients, particularly proteins, which are indispensable for human health. Pork, ranking second in global meat consumption, provides over a quarter of the world's total protein intake [1]. Despite a four-fold increase over the past 50 years, traditional meat production is nearing its capacity and may struggle to meet the rising demand [2]. Moreover, conventional farming systems give rise to several environmental and public health issues, including greenhouse gas emissions, excessive water and land use, the spread of zoonotic diseases, and concerns regarding animal welfare [3].

In response, researchers are diligently exploring sustainable alternatives to traditional meat. Among these, plant-based meat analogues and animal cell-cultured meat (CM) have captured significant attention [4]. Plant-based meat has been explored since 1888 and holds potential for widespread acceptance, including among vegetarian and halal diets. However, it often fails to replicate the texture, flavor, and complete nutritional profile of real meat, particularly in terms of amino acid composition [5]. In contrast, CM closely mimics actual meat by producing real muscle tissue, and advanced significantly since the debut of the first cultured hamburger in 2013 [6]. This progress has been driven by breakthroughs in stem cell biology and tissue engineering, particularly the use of muscle stem cells (MuSCs). MuSCs, which reside quiescently between muscle sarcolemma and basal lamina, can be activated to repair muscle tissue upon injury [7]. Given that meat's fundamental structure consists of bundles of striated myofibers, which are essential for its sensory and nutritional properties [8], replicating this structure through MuSCs differentiation and alignment is a key focus of CM research.

Nevertheless, CM production is still in its infancy. A major challenge lies in replicating the texture and nutritional attributes of conventional meat. Cells are highly niche-dependent, and scaffolding is pivotal for creating a niche-like environment that supports tissue geometry and cell distribution. Ideal CM scaffolds must be edible, biocompatible, cost-effective, and readily available, while also facilitating cell adhesion, myofiber alignment and maturation, and supporting extracellular matrix (ECM) production [8]. However, current synthetic scaffolds risk generating toxic byproducts, and animal-derived materials like collagen and gelatin, though safe, are often costly and non-renewable [3,8]. This has prompted a shift towards renewable plant-based materials, which offer sustainability, biocompatibility, and the potential to replicate the structure of conventional meat. Examples include plant proteins [[9], [10], [11], [12]], plant polysaccharides [13], plant leaf veins [[14], [15], [16]], and plant-based composites [17]. Despite extensive research on plant-derived scaffolds for CM, challenges persist, including limited mechanical strength and the need for chemical processing. Additionally, while aligned scaffolds have been shown to enhance myotube formation and maturation compared to non-organized structures [18], accurately replicating the complex microstructure of muscle tissue remains a significant challenge.

To overcome these limitations, we explore the feasibility of using lotus fiber as a novel plant-based scaffold for CM production. Lotus fiber, derived from lotus roots and petioles, is primarily composed of cellulose, hemicellulose, lignin, and pectin [19]. Specifically, cellulose and pectin are recognized by the FDA as dietary fiber components [20]. Additionally, the FDA's 2018 guidance on dietary fiber declaration in nutrition and supplement labeling reinstated “mixed plant cell wall fibers” as dietary fiber, which includes lignin as a natural component of plant cell walls [21]. Although hemicellulose is not explicitly listed, it is implied as part of the “mixed plant cell wall fibers.” These components ensure the biocompatibility and edibility of lotus fiber. Furthermore, this scaffold features a highly aligned, bundled fiber structure with superior mechanical properties that closely emulates the complex architecture and integrity of traditional meat, making it ideally suited for muscle cell alignment [22]. Considering its edible nature, structural attributes, mechanical properties, and abundance, lotus fiber is a promising scaffold for CM production.

In the present study, we developed a CM that mirrors the essential food-related characteristics and nutritional values of traditional meat, harnessing the untapped potential of a novel lotus fiber-based natural plant fiber (NPF) scaffold. We assessed its morphology, mechanical properties, and chemical composition for CM suitability. Subsequently, we evaluated its effects on porcine MuSCs (pMuSCs), including cell viability, proliferation, morphological changes, and myogenic differentiation in response to fiber alignment. The differentiated cells were characterized at both the transcriptome and proteome levels to provide a comprehensive analysis of the CM. Additionally, we assessed the food-related characteristics of the final product, including visual appearance, texture properties, and nutritional values. Notably, the NPF scaffolds also facilitated the differentiation of porcine adipose-derived stem cells (pADSCs) into lipid droplet-containing adipocytes, which are crucial for meat juiciness, tenderness, and flavor. Our findings demonstrate the NPF scaffold's potential in CM production, particularly in enriching the product's food characteristics. By introducing a non-animal-origin, food-grade, and low-cost biomimetic scaffold, our research offers a facile strategy for sustainable food production.

## 2 Results

### 2.1 Preparation and characterization of the NPF scaffold

**Fig. 1:**
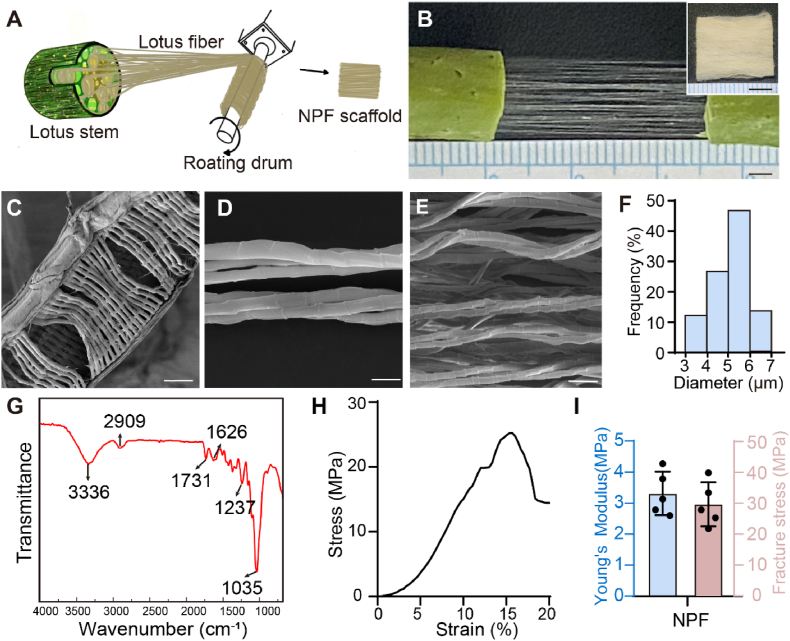
Preparation and characterization of the NPF scaffold. A) Schematic representation of the NPF scaffolds preparation process. B) Images of lotus fiber extracted from the lotus stem. Scale bars = 3 mm. The insert showing the overall morphology of the NPF scaffold. Scale bars = 5 mm. C-D) SEM images depicting the microstructure of lotus fiber bundles within the lotus stalk (C), a longitudinal view of the fiber bundles (D). Scale bars represent 50 μm (C) and 10 μm (D). E) The microtopography of the NPF scaffolds. Scale bars = 20 μm. F) Histogram showing the diameter distribution of lotus fibers (n = 200). G) FTIR spectra of the NPF scaffold. H) Stress-strain curve of the NPF scaffold. I) Mechanical properties of the NPF scaffold, including Young's modulus and fracture stress (n = 5). Results are presented as mean ± SD.

The fabrication of the NPF scaffolds began with the gentle breaking of lotus petioles, followed by the extraction of lotus fibers by applying opposing tension at both ends. The fibers were then aligned and uniformly wrapped them around a rotating drum to form a coherent structure. Finally, the fiber mats were carefully detached from the drum for further use (Fig. 1A and B). The well-organized structure of scaffolds is crucial for directing cell orientation and behaviors. SEM characterization showed that the lotus fibers are orderly assembled within the vascular bundle with a unique ribbon-like spiral structure (Fig. 1C and Fig. S1A). These fibers, composed of several monofilaments, are closely associated, forming a structured helical arrangement (Fig. S1B). Physical stretching of these fibers tends to weaken their spiral configuration (Fig. 1D). SEM images of the fiber mats confirmed a high degree of fiber alignment (Fig. 1E), and the cross-section of lotus fiber is typically elliptical or nearly circular (Fig. S1C), with a fineness ranging from 3 μm to 7 μm (Fig. 1F). The mean diameter of the fiber is around 5.06 ± 0.89 μm, which is comparable to the diameter range of smooth muscle fibers in pigs, typically 2–10 μm [23].

The non-toxic and edible qualities of the NPF scaffolds are fundamental for its use in CM applications. Chemical composition analysis of the NPF scaffolds were conducted using an FTIR spectrometer (Fig. 1G). The spectra indicated the presence of O-H or N-H stretching vibrations at 3336 cm^−1^, indicating hydroxyl and amide groups; the CH- and CH_2_- stretching at 2909 cm^−1^, characteristic of aliphatic chains; C=O stretching at 1731 cm^−1^, associated with pectin esterase, carboxylic acid, or hemicellulose acetyl groups; the lignin oxygen group at 1626 cm^−1^; the C-O vibration in galactan and hemicellulose at 1237 cm^−1^, and the C-O vibration in polysaccharide cellulose at 1035 cm^−1^, respectively [19]. These spectral features are consistent with previous research that identified cellulose as the predominant component of lotus fiber [24]. Cellulose is well-known for its dietary fiber benefits, playing a significant role in gut health and influencing the microbiome [25]. Additionally, lotus fiber contains hemicellulose, lignin, fat waxy, pectin, and amino acids [19]. Although lotus fibers lack ECM components, the functional groups in these natural polysaccharides and cellulose structures (such as the hydroxyl groups) can promote cell attachment through non-specific interactions (e.g., hydrogen bonds and van der Waals forces). Unlike ECM protein-mediated adhesion (e.g., collagen), this mechanism relies on physicochemical interactions between the scaffold's surface chemistry and the cell membrane [26,27]. To further verify the role of cellulose hydroxyl groups in cell adhesion, we characterized the NPF scaffold's wettability and surface charge (zeta potential). The water contact angle (88.2 ± 8.2°), reflects the scaffold's hydrophilicity, attributable to surface-exposed hydroxyl groups (-OH) that form hydrogen bonds with water molecules (Fig. S1D). Additionally, the measured zeta potential value was −9.4 ± 0.4 mV, which forms a moderate electrostatic attraction with the cell surface charge, thereby providing favorable conditions for cell attachment (Fig. S1E). This highlights the scaffold's potential in cell culture applications [28].

The mechanical properties of the NPF scaffolds are another critical factor, as they directly influence the organoleptic properties of the final food product. The typical tensile stress-strain curve and Young's modulus of the lotus fiber demonstrated its high strength and low extensibility (Fig. 1H and I). This characteristic is attributed to the spiral structure, which enables the lotus fiber to be highly stretchable and capable of withstanding large strain without fracturing. Together with its high fiber content, parallel architecture, appropriate diameter, commendable mechanical strength, and low crystallinity [24], these features collectively indicate lotus fibers as an ideal candidate for CM scaffolds.

### 2.2 Proliferation and morphology of pMuSCs on NPF scaffolds

To evaluate the influence of fibers on the pMuSCs behaviors, the cells were first isolated and characterized. After one week of *ex vivo* culture, the pMuSCs exhibited increased size and adopted a spindle-like shape, as indicated by the elevated aspect ratios and mean areas (Fig. S2A and S2B). Immunofluorescent staining for PAX7, a marker critical for identifying functional satellite cells [29], revealed that nearly 83 % of the cells were positive for this marker, signifying a myogenic precursor population (Fig. S2C). Flow cytometry was employed to further authenticate the pMuSCs identity by assessing the expression of CD29 and CD56, satellite cell-specific markers [29]. The analysis indicated that 89.2 % of the cells co-expressed CD29 and CD56, corroborating the myogenic and stem cell properties of the isolated cells (Fig. S2D).

**Fig. 2:**
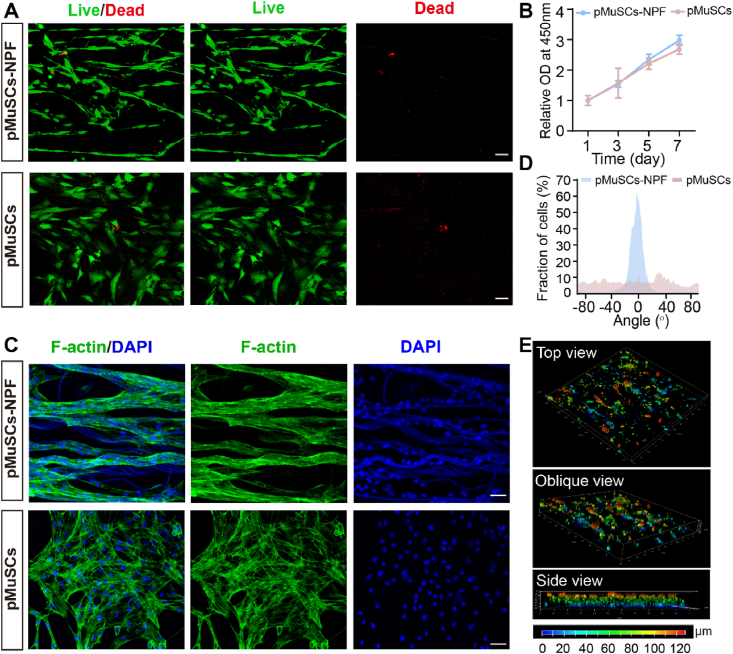
Cytocompatibility of pMuSCs cultured on the NPF scaffold. A) Live/dead cell staining of pMuSCs cultured on the NPF scaffolds and plate (green fluorescence indicates live cells, while red fluorescence indicates dead cells). Scale bars = 50 μm. B) Proliferation of pMuSCs cultured on the NPF scaffolds and plates at day 1, 3, 5, and 7 (n = 5). C) Fluorescence images of F-actin staining showing the morphology of pMuSCs cultured on the NPF scaffolds and plate. Scale bars = 50 μm. D) Histogram showing the distribution of cell orientation angles for pMuSCs cultured on the NPF scaffolds and plates. E) Three-dimensional reconstructions of DiI-labeled pMuSCs within the NPF scaffold, viewed from top, oblique, and side perspective. A scale bar of 100 μm is included for reference. Results are presented as mean ± SD.

The biocompatibility of the NPF scaffolds were assessed using a live/dead staining assay on pMuSCs at 3 days post-seeding. The results demonstrated high viability for pMuSCs on both the NPF scaffolds (pMuSCs-NPF) and on the plastic plates (Fig. 2A). Cell density analysis over time confirmed cell proliferation within the scaffold, comparable cell viability and proliferation rates to those observed on plastic plates (Fig. 2B). The alignment of muscle cells is a pivotal factor in the initial phases of *in vitro* meat production, as it is integral to the development and maturation of muscle fibers [30]. F-actin staining revealed pronounced elongation and unidirectional alignment of pMuSCs cultured on the NPF scaffold, whereas conventional plastic plates which failed to induce anisotropic signals, resulting in a disorganized cellular arrangement (Fig. 2C and D). This preferential alignment on the NPF scaffolds is likely to promote the formation of organized muscle fibers. In addition, a comprehensive 3D analysis of DiI-stained pMuSCs demonstrated even infiltration and distribution of pMuSCs throughout the scaffold (Fig. 2E). Collectively, these results demonstrated the NPF scaffold's excellent cytocompatibility, high cell viability, and its capacity to promote cell proliferation, alignment and migration, suggesting its potential for use in CM production.

### 2.3 NPF scaffolds enhance the myogenesis in pMuSCs

**Fig. 3:**
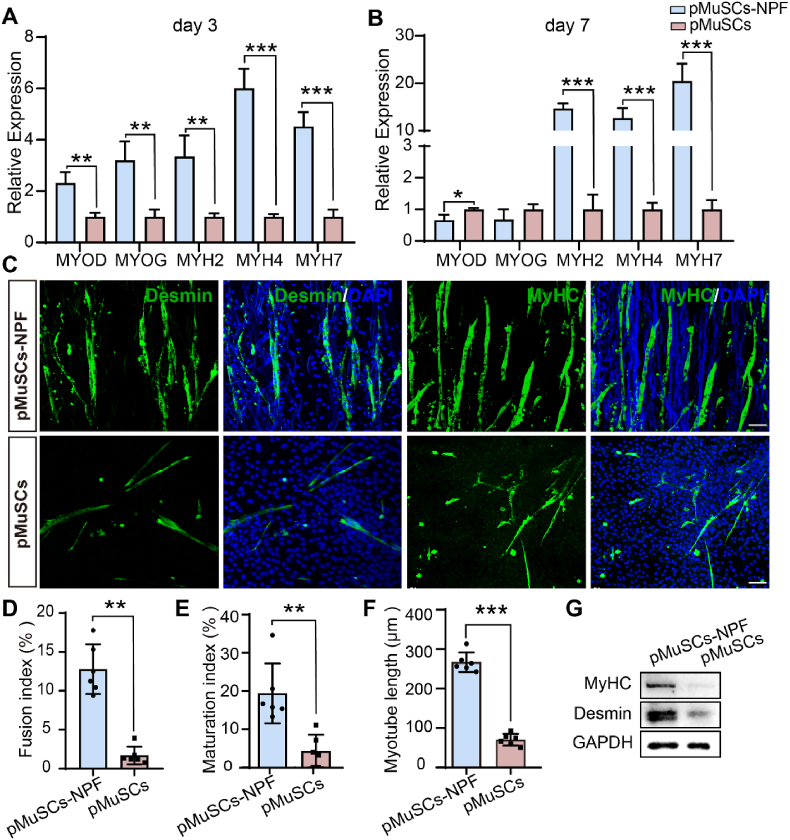
Enhanced myogenic differentiation and myotube formation of pMuSCs on NPF scaffolds. A, B) RT-qPCR analysis showing expression levels of MYOD, MYOG, MYH2, MYH4, and MYH7 in pMuSCs cultured on NPF scaffold and plate after 3 and 7 days, respectively. The fold change is normalized to the pMuSCs control group (n = 3). C) Immunofluorescence images of Desmin and MyHC expression in the pMuSCs-NPF and pMuSCs group. Scale bars = 50 μm. D-F) Quantitative assessment of myotube formation, including the fusion index (D), maturation index (E), and myotube length (F) (n = 6). G) Representative Western blot analysis of Desmin and MyHC protein levels in pMuSCs-NPF and pMuSCs groups. Results are presented as mean ± SD, statistical comparison was performed by a two-tailed *t*-test. ∗*p* < 0.05, ∗∗*p* < 0.01, ∗∗∗*p* < 0.001.

The correlation between cell shape and cell function implies that the NPF scaffolds may potentially promote myogenic differentiation in pMuSCs. To validate this hypothesis, we induced myogenesis and assessed the expression of key myogenic genes and proteins. Our mRNA expression analysis focused on key myogenic markers: myoblast determination protein 1 (MYOD, an early-stage marker); myogenin (MYOG), an intermediate-stage marker; and members of the MyHC (myosin heavy chain) family (MYH2, MYH4 and MYH7), which indicate the maturation and terminal differentiation of myocytes [31]. After 3 days, pMuSCs cultured on NPF scaffolds exhibited significantly upregulation of MYOD (2.32-fold, *p* < 0.01)*,* MYOG (3.21-fold, *p* < 0.01)*,* MYH2 (3.35-fold, *p* < 0.01)*,* MYH4 (6.01-fold, *p* < 0.001)*,* and MYH7 (4.52-fold, *p* < 0.001) compared to those on plastic plates (Fig. 3A). At 7 days post-induction, pMuSCs-NPF group exhibited reduced MYOD (0.66-fold, *p* < 0.05) and MYOG (0.68-fold)*,* with concurrent elevated levels of MYH2 (14.69-fold, *p* < 0.001)*,* MYH4 (12.69-fold, *p* < 0.001)*,* and MYH7 (20.45-fold, *p* < 0.001) compared to the pMuSCs group (Fig. 3B). The observed decrease in MYOD and MYOG, together with the increased MyHC gene expression at day7, suggests accelerated myotube maturation on NPF scaffolds.

Muscle regulatory factors activate MuSCs, promoting their differentiation into myoblasts, which subsequently fuse to form myotubes. Desmin, a muscle-specific filament protein, plays a crucial role in myogenesis and muscle formation [32]. Immunofluorescence revealed enhanced Desmin and MyHC expression in pMuSCs on NPF scaffolds (Fig. 3C). After a 7-day differentiation, pMuSCs cultured on NPF scaffolds formed longer and denser myotubes compared to the pMuSCs control, which is indispensable for structured meat production. This enhancement was quantified by a higher nuclei fusion index (12.8 ± 3.2 % *vs* 1.7 ± 1.1 %, *p* < 0.01), maturation index (19.4 ± 7.8 % *vs* 4.2 ± 4.3 %, *p* < 0.01), and myotube length (267.4 ± 24.8 μm *vs* 70.6 ± 14.9 μm, *p* < 0.001) in the NPF scaffolds group (Fig. 3D–F). Additionally, multinucleated myotubes on the NPF scaffolds aligned with the fiber direction, unlike the disorganized differentiation on plates. In line with these findings, Western blot analysis showed a 2.1-fold (*p* < 0.05) and 5.4-fold (*p* < 0.05) increase in Desmin and MyHC levels in pMuSCs-NPF, respectively, compared to the pMuSCs group (Fig. 3G, and Fig. S3A and S3B). Collectively, these findings demonstrate that the NPF scaffolds guide the pMuSCs elongation and alignment, enhancing myogenic differentiation and myotubes formation, potentially improving CM production quality.

### 2.4 Transcriptomics of pMuSCs differentiated on NPF scaffolds: insights into myogenesis and ECM remodeling

**Fig. 4:**
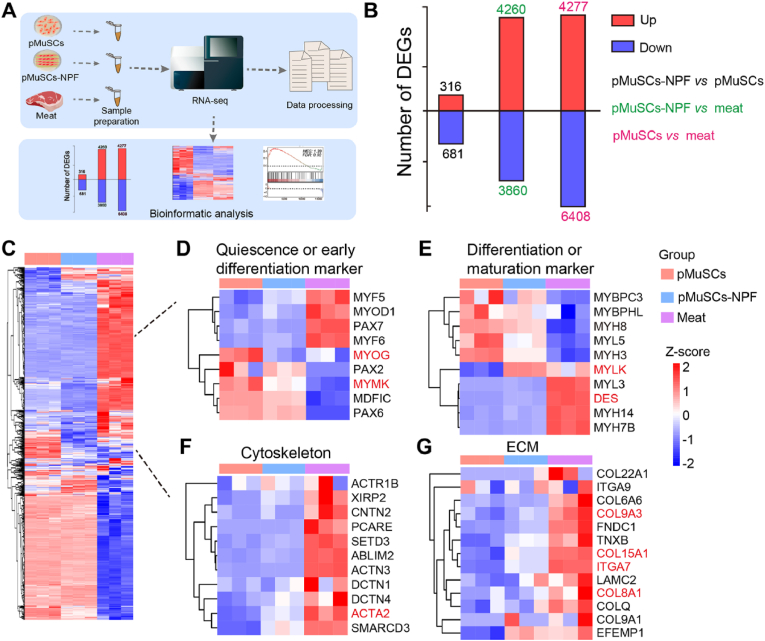
Comprehensive transcriptomic analysis of pMuSCs, pMuSCs-NPF, and meat. A) Schematic overview of the experimental design for transcriptomics profiling. B) Bar graph illustrating the number of differentially expressed genes (DEGs) identified in pairwise comparisons: pMuSCs-NPF *vs* pMuSCs, pMuSCs-NPF *vs* meat, and pMuSCs *vs* meat. C) Heatmap depicting the DEGs related to muscle development across the three sample groups. D-G) Heatmaps of DEGs associated with distinct myogenic processes: quiescence or early differentiation markers (D), differentiation or maturation markers (E), cytoskeleton (F), and ECM (G). Key genes are highlighted in red. MYOG and MYMK are essential for early myogenesis, while DES, MYLK, and ACTA2 contribute to the development of mature muscle structure and regulate cell adhesion, migration, morphology, and contractility. ECM components (ITGA7, COL9A3, COL15A1, and COL8A1) play vital roles in muscle cell physiology.

To elucidate the intricate mechanisms underlying the activation and differentiation of pMuSCs on NPF scaffolds, we performed RNA-sequencing (RNA-seq) to compare the transcriptomes of pMuSCs cultured on NPF scaffolds with those cultured on traditional plates. Additionally, we benchmarked these against the gene expression patterns observed in authentic muscle tissue, focusing particularly on myogenic differentiation markers (Fig. 4A). After a 7-day culture, three biological replicates of pMuSCs, pMuSCs-NPF, and real meat were subjected to RNA-seq. Differentially expressed genes (DEGs) were identified with a threshold of at least two-fold change and a false discovery rate (FDR) of less than 0.05. Specifically, the pMuSCs-NPF group exhibited 316 upregulated and 681 downregulated DEGs compared to the pMuSCs group. In the comparison of pMuSCs-NPF versus meat, 4260 genes were upregulated, whereas 3860 genes were downregulated. Similarly, the comparison between pMuSCs and meat revealed 4277 upregulated and 6408 downregulated DEGs (Fig. 4B). These results highlight significant gene expression differences associated with the culture conditions and muscle tissue maturation. Among these DEGs, we observed substantial variations in genes essential for muscle development (Fig. 4C). A heatmap graphically represented the relative expression levels of key myogenic differentiation genes, including those associated with satellite cells quiescence and early differentiation markers, differentiation and maturation markers, cytoskeleton markers, and ECM markers (Fig. 4D–G). For example, MYOG and MYMK were downregulated in pMuSCs-NPF and meat compared to pMuSCs. These genes are integral to the early myogenesis and typically decrease following myotube formation [33]. In contrast, DES, MYLK, and ACTA2 are essential for the development of mature muscle structure, as well as for cell adhesion, migration, shape regulation, and contractility [34,35], were notably upregulated in pMuSCs-NPF and meat compared to pMuSCs. Additionally, MuSCs differentiation is accompanied by ECM component deposition, which play an important role in the physiological functions of muscle cells [36]. In accordance with this, ECM-related genes were upregulated in pMuSCs-NPF and meat, including ITGA7, COL9A3, COL15A1, and COL8A1. Specifically, ITGA7, an integrin receptor, plays a central role in maintaining muscle function and structure [37]. To confirm the accuracy of our transcriptome sequencing data and support the biological relevance of the identified differentially expressed genes. We validated several differentially expressed genes associated with myogenic differentiation using RT-PCR. The RT-PCR results were highly consistent with the trends observed in the transcriptome sequencing data. For example, the expression levels of quiescence or early differentiation markers, such as MYOG and MYMK, were lower in pMuSCs-NPF and meat compared to pMuSCs. In contrast, markers associated with differentiation or maturation, such as DES, cytoskeletal markers like ACTA2, and ECM-related markers like ITGA7, exhibited higher expression levels in pMuSCs-NPF and meat than in pMuSCs. The observed expression levels revealed statistically significant differences (Fig. S4).

We further investigated the signaling pathways and biological processes activated in pMuSCs cultured on the NPF scaffolds. gene ontology (GO) enrichment analysis revealed significant enrichment of upregulated genes in biological processes like cell differentiation and intercellular communication, and molecular functions such as ion channel activity and receptor binding (Fig. S5A). Kyoto Encyclopedia of Genes and Genomes (KEGG) analysis indicated the involvement of upregulated genes in myogenesis-related pathways (Fig. S5B). Gene Set Enrichment Analysis (GSEA) was employed to further investigate the biological processes in pMuSCs-NPF, showing significant enrichment of ECM proteoglycans, glycoproteins, and structural constituents compared to pMuSCs (Fig. S6A). A circular heatmap illustrating the DEGs based on GSEA results is shown in Fig. S6B. ECM genes crucial for muscle regeneration, like COL1A2, COL12A1, and fibronectin (FN1), were upregulated in pMuSCs-NPF, indicating ECM's role in myotube formation and highlighting NPF scaffolds' potential to modulate ECM organization [36]. Together, our RNA-seq analysis indicates that NPF scaffolds significantly impact the transcriptome of pMuSCs, activating genes associated with muscle development and ECM remodeling. This activation propels myogenesis in pMuSCs and aligns the transcriptomic profile of pMuSCs-NPF closer to real meat, suggesting the potential of NPF scaffolds for CM applications.

### 2.5 Proteomic characteristics of pMuSCs differentiated on NPF scaffolds

**Fig. 5:**
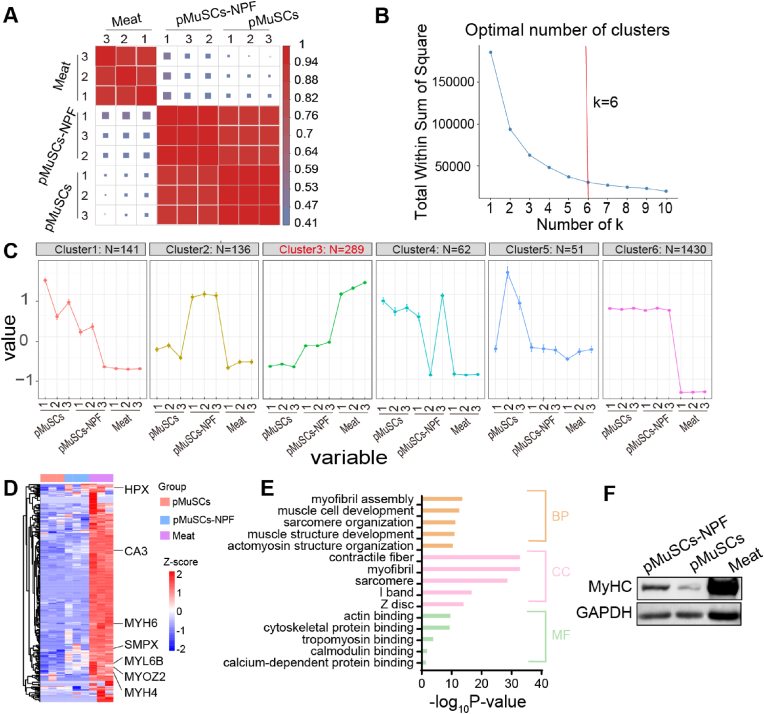
Proteomic clustering and functional analysis of pMuSCs, pMuSCs-NPF, and meat. A) Correlation heatmap shows the relationship among the replicates of pMuSCs, pMuSCs-NPF and meat. B) Scatter plot used to determine the optimal number of clusters (*k*). C) A total of six clustering categories were created based on differential protein expression patterns in pMuSCs, pMuSCs-NPF, and meat groups. Each cluster represents a unique set of proteins with similar expression profiles. D) Heatmap of the proteins within cluster 3. E) Enriched GO terms associated with cluster 3, categorized by BP, CC, and MF, which are related to muscle development, organization, and function. F) Western blot analysis confirming the differential expression of the MyHC protein among pMuSCs, pMuSCs-NPF, and meat.

Given that cellular differentiation process entails alterations in both gene expression and protein profiles, we conducted a proteomic analysis 7 days post-induction using a liquid chromatography-tandem mass chromatography (LC-MS/MS) system. A total of 3696 proteins were identified, with their correlations graphically represented in a heatmap (Fig. 5A). A comparative proteomic analysis was conducted to elucidate differential protein expression patterns among pMuSCs, pMuSCs-NPF, and meat. Utilizing the k-means clustering algorithm, we categorized differentially expressed proteins (DEPs) into six distinct clusters, which may correspond to specific biological pathways or mechanisms (Fig. 5B and C). Each cluster displayed unique proteomic alterations, with cluster 3 showing a pattern closely associated with myogenic differentiation, as depicted in the heatmap (Fig. 5D). Myogenic-associated proteins exhibited a progressive increase in expression from pMuSCs to pMuSCs-NPF and finally to meat. Notably, myosin-related proteins, such as MYH4, MYH6, and MYL6B, which are important components of mature muscle structure [38], showed a gradient of expression: low in pMuSCs, moderate in pMuSCs-NPF, and high in meat. This pattern was also observed in proteins like SMPX, which is implicated in maintaining muscle integrity [39], and potential meat tenderness markers HPX and MYOZ2 [40], as well as the abundant muscle protein carbonic anhydrase III (CA3) (Fig. 5D) [41]. Furthermore, GO term functional enrichment analysis of cluster 3 highlighted significant changes in DEPs related to muscle development, organization, and function. For instance, biological processes (BP) terms associated with cluster 3 are crucial for muscle formation, organization, and function. In the cellular component (CC) category, terms like “contractile fiber”, “myofibril”, “sarcomere”, “I band”, and “Z disc” are all vital to muscle cell structure and function. The molecular function (MF) terms primarily involve processes related to muscle structure and cell development, ranging from the construction of the overall muscle architecture to the development of muscle cells (Fig. 5E). Collectively, these GO terms highlight key processes and structural aspects of muscle development, organization, and function. Our findings suggest that pMuSCs-NPF exhibit characteristics that are intermediate between pMuSCs and meat. Specifically, there is a progressive increase in the expression of proteins associated with mature myoblasts and myofibers, from pMuSCs to pMuSCs-NPF and finally to meat. Western blot analysis of MyHC protein expression in pMuSCs-NPF, pMuSCs, and meat further supports our proteomic findings (Fig. 5F). These results suggest that the NPF scaffolds may enhance the maturation and muscle-like characteristics of pMuSCs.

### 2.6 Proteomic analysis unveils enhanced maturation in pMuSCs on NPF scaffolds

**Fig. 6:**
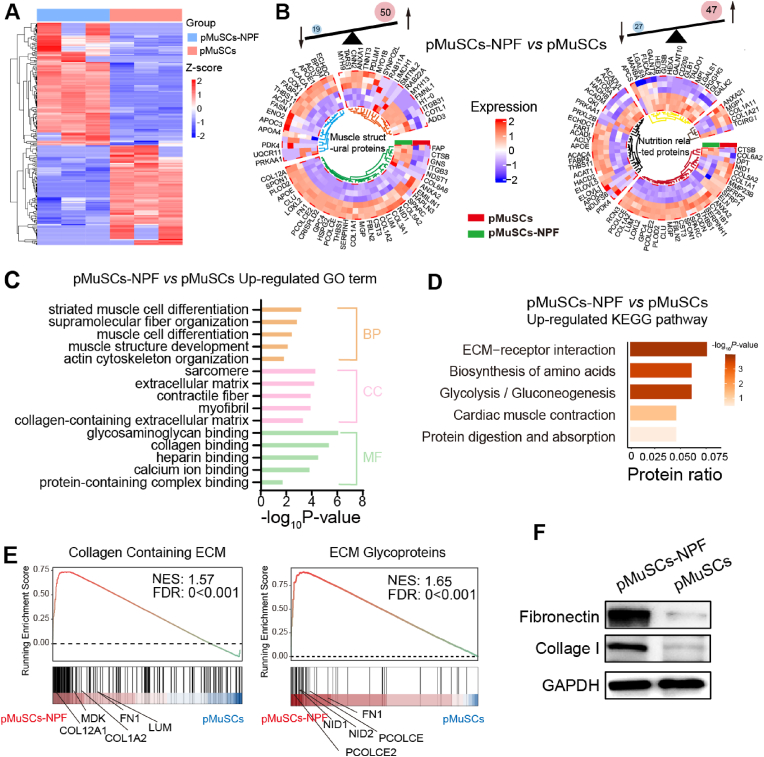
Comparative proteomic analysis of pMuSCs-NPF and pMuSCs. A) Heatmap of differential expression proteins (DEPs) between pMuSCs-NPF and pMuSCs. B) Circular heatmap summarizing the DEPs categorized into muscle structural proteins (left) and nutrition-related proteins (right) between pMuSCs-NPF and pMuSCs. Each color represents a different protein class: myofibrillar (orange), sarcoplasmic (blue), stroma (green), mineral and vitamin-associated (brown), carbohydrates-associated (yellow), fatty acid-associated (black), and collagen-associated (red) proteins. C) GO enrichment analysis of the significantly altered protein set associated with muscle development and organization between pMuSCs-NPF and pMuSCs. D) KEGG pathway enrichment analysis of the significantly altered proteins sets involved in muscle function and structure between pMuSCs-NPF and pMuSCs. E) GSEA plots depicting the enrichment of collagen containing ECM and ECM glycoproteins in pMuSCs-NPF compared to pMuSCs. NES = normalized enrichment score; FDR = false discovery rate. F) Western blot analysis of fibronectin and collagen I protein expression levels in pMuSCs-NPF and pMuSCs groups.

The nutritional profile of CM has not been extensively explored in existing research. To gain a deeper understanding of the proteomics of pMuSCs-NPF, we conducted a comprehensive analysis of DEPs between pMuSCs-NPF and pMuSCs. Our analysis identified 277 DEPs with significant two-fold changes (FDR <0.05), indicating robust cellular response induced by the NPF scaffolds in pMuSCs (Fig. 6A). These DEPs were systematically categorized into two functional clusters: one associated with muscle structure and the other with nutritional components (Fig. 6B). The muscle structure cluster includes stroma proteins, sarcoplasmic proteins, and myofibrillar proteins [42], comprising 50 upregulated and 19 downregulated proteins. These proteins are rich in essential amino acids, which are vital for the body's efficient absorption and utilization. They also significantly contribute to the meat's water-holding, gelation, and emulsification properties, influencing its mouthfeel [43]. The nutrition-related cluster includes 47 upregulated and 27 downregulated proteins. These proteins were classified based on human nutritional requirements [44]. This cluster included proteins linked to collagen, fatty acids, carbohydrates, minerals, and vitamins, reflecting CM's intricate nutritional profile.

GO enrichment analysis was performed to probe the cellular processes influenced by the NPF scaffold. BP terms such as “striated muscle cell differentiation”, “supramolecular fiber organization”, “muscle cell differentiation”, “muscle structure development”, and “actin cytoskeleton organization”, are fundamental for muscle cell formation, tissue construction, and the orderly arrangement of intracellular structures. CC terms such as “sarcomere”, “extracellular matrix”, “contractile fiber”, “myofibril”, and “collagen-containing extracellular matrix”, are critical for muscle cell structure and function, as well as for maintaining muscle contractility and tissue organization. MF terms relate to molecular binding functions, which are essential for various physiological processes, including extracellular matrix organization, signal transduction, substance transport, and cell structure maintenance (Fig. 6C), all these elements are crucial for muscle development. Subsequently, KEGG enrichment analysis identified activated signaling pathways in pMuSCs-NPF compared to pMuSCs. Results showed significant pathway activation in pMuSCs-NPF, such as “ECM-receptor interaction”, “cardiac muscle contraction”, “glycolysis/gluconeogenesis”, “biosynthesis of amino acids”, and “protein digestion and absorption”. These pathways are crucial for energy production, as well as for the synthesis and metabolism of proteins (Fig. 6D). GSEA confirmed the enrichment of “collagen containing ECM” and “ECM glycoproteins” in pMuSCs-NPF (Fig. 6E). Among these, fibronectin, a key structural element in the stem cell niche, is critical for MuSCs maintenance and function during muscle regeneration [45]. It promotes earlier alignment and fusion of cells compared to laminin or gelatine, facilitating myoblast fusion and elongation. Fibronectin acts through integrins on myoblasts, and N-cadherin contributes to myoblast alignment [46]. Accordingly, our proteomic analysis revealed a significant upregulation of cadherin proteins CDH11 (2.2-fold, *p* < 0.05) and CDH13 (1.7-fold, *p* < 0.05) in pMuSCs-NPF compared to pMuSCs (Fig. S7A and S7B). Additionally, collagen I, a major ECM component, is known to facilitate myogenesis and serve as an additional protein source for CM [47]. Western blot assay confirmed increased levels of fibronectin (5.1-fold, *p* < 0.05) and collagen I (2.7-fold, *p* < 0.05) in pMuSCs cultured on NPF scaffolds, attributing to enhanced myogenesis (Fig. 6F, and Fig. S7C and S7D).

In conclusion, our comprehensive omics analyses demonstrate the NPF scaffold's superior ability to enhance myotube maturation and ECM deposition compared to conventional plastic plates, creating a favorable environment for CM development. Our study provides an in-depth examination of scaffolds that promote differentiation and compares them with traditional meat, thereby contributing to understanding the differences and similarities between cultured meat and traditional meat.

### 2.7 Characterization of cultured meat prototype: appearance, sensory, and nutritional profiles

**Fig. 7:**
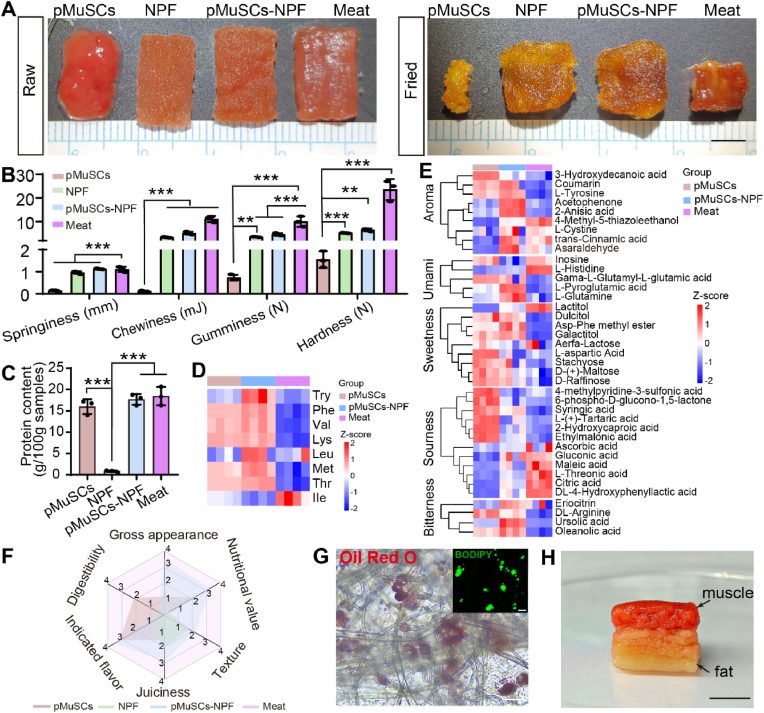
Comprehensive assessment of cultured meat engineered on NPF scaffolds. A) Representative images of raw and cooked samples from pMuSCs, NPF, pMuSCs-NPF, and meat. B) TPA of pMuSCs, NPF, pMuSCs-NPF, and meat, including springiness, chewiness, gumminess, and hardness (n = 3). C) The protein content per 100 g for pMuSCs, NPF, pMuSCs-NPF, and meat (n = 3). D) Heatmap illustrating the levels of essential amino acids in pMuSCs, pMuSCs-NPF, and meat. E) Heatmap of indicated flavor in pMuSCs, NPF, pMuSCs-NPF, and real meat, including aroma, umami, sweetness, sourness, and bitterness. F) Radar map summarizing the sensory evaluation scores across multiple dimensions: gross appearance, nutritional value, texture, juiciness, indicated flavor, and digestibility for pMuSCs, NPF, pMuSCs-NPF, and meat (n = 3). G) Oil Red O and BODIPY immunofluorescence staining of lipid droplets in pADSCs cultured on NPF scaffolds. Scale bars = 50 μm. H) Photographic depiction of a centimeter-scale assembled pork belly prototype. Scale bars = 2 cm. Results are presented as mean ± SD, One-way ANOVA with Tukey's multiple comparison tests were used. ∗∗*p* < 0.01, ∗∗∗*p* < 0.001.

After confirming changes in the myogenesis, the visual appearance and texture attributes of CM, key sensory determinants that influence consumer acceptance were analyzed. In terms of the appearance of raw and cooked samples (Fig. 7A), pMuSCs-NPF closely resembled real meat with a vibrant red color and a glossy surface. Upon cooking, all samples turned yellowish brown due to the Maillard reaction. It is crucial for cooked meat products to maintain juiciness and minimize water loss during cooking. In the case of pure pMuSCs, they shrunk significantly after cooking, whereas other groups retained their appearance well. Moisture content analysis revealed comparable water content among pMuSCs, pMuSCs-NPF, and meat, but pMuSCs alone suffered considerable water loss (∼75 %) after cooking. In contrast, both the pMuSCs-NPF and meat exhibited superior water holding capacity (Fig. S8A and S8B). Mechanical properties are essential for CM to mimic the sensory characteristics of conventional meat [6]. The current CM prototypes face the challenge of lacking firmness, which affects the natural chewing sensation. Mouthfeel largely relies on the elasticity of the structures. Rheological characterization showed that the storage modulus (G′) values were consistently higher than the loss modulus (G″) values for all samples, indicating solid-like properties. Notably, pMuSCs-NPF and NPF had storage modulus values similar to natural meat (G' ∼11 kPa), while pMuSCs alone had significantly lower values (G' < 100 Pa), suggesting a lack of structural support from scaffolds (Fig. S8C and S8D). Furthermore, the TPA was performed to assess hardness, springiness, chewiness, and gumminess of all samples. The pMuSCs-NPF exhibited a tenderer texture compared to traditional meat, but their springiness was similar. Whereas pMuSCs without scaffolds markedly differed from traditional meat (Fig. 7B). This disparity could be attributed to the absence of the fibrous bundle structure characteristic of muscle tissue in the accumulated cells.

To fundamentally address the longer-term challenge of sustainably producing protein alternatives, CM products should match the nutritional content of livestock meat. Protein content is a key nutritional indicator for meat quality. The protein content in pMuSCs-NPF was comparable to that in conventional meat and significantly higher than that of bare NPF scaffolds (Fig. 7C). Collagen, a primary component of the muscle extracellular matrix, plays a crucial role in providing structural support and facilitating cellular connections [36]. The collagen content in pMuSCs-NPF was significantly higher than in pure cells or bare NPF, which is expected to enhance the elasticity and chewiness of CM (Fig. S8E). It also illustrates that while the pure NPF looks like meat, cells cultured on the NPF scaffolds are still required to achieve its nutritional value. Amino acid profile is another crucial nutritional indicator of meat, particularly essential amino acids, which the body cannot synthesize and must obtain through diet [48]. Untargeted metabolomics revealed elevated levels of seven out of eight essential amino acids in both the pMuSCs-NPF and pMuSCs groups compared to real meat, with particularly notable increases observed in the pMuSCs-NPF group (Fig. 7D). These findings confirmed that the NPF scaffold's regulation of cell differentiation significantly influences the nutritional profiles of the final CM. In addition, the amino acid composition of meat is pivotal in dictating its taste sensations, which are intricately linked to a spectrum of basic flavors. These include sourness, sweetness, saltiness, bitterness, and umami, all of which contribute to the complex taste experience of meat [49]. Therefore, to replicate the sensory attributes of traditional meat, CM must closely mimic this amino acid composition. We analyzed the flavor-related amino acids in pMuSCs, pMuSCs-NPF, and meat (Fig. 7E). In the Maillard reaction, it initiates a series of reactions that generate aromatic compounds, which are crucial for forming the distinctive flavor of meat [50]. Notably, 4-methyl-5-thiazoleethanol, a sulfur-containing heterocyclic compound plays a significant role in the meat aroma formation during the Maillard reaction, imparting distinct roasted and burnt aroma that enhance the richness and uniqueness of the meat aroma [51]. Umami attribute of meat is important for good taste. Glutamic acid serves as the primary umami contributor [52], binding to umami taste receptors and triggering the perception of umami taste. L-Glutamine is a non-essential amino acid, can be converted into L-glutamic acid through a series of metabolic processes in the body. Once L-glutamine is converted into L-glutamic acid, it becomes crucial not only not only for natural meat flavor development but also for enhancing umami in processed foods and additives like monosodium glutamate [53]. Additionally, L-pyroglutamic acid, a cyclic glutamic acid derivative, possesses a distinct umami flavor. Studies demonstrate its role as an active flavor component in fermented foods such as kimchi, where it contributes salty, umami, and sour notes, potentially through direct interaction with umami receptors [54]. Our analysis showed that pMuSCs-NPF contained the highest levels of L-glutamine and L-pyroglutamic acid, suggesting enhanced umami potential compared to conventional meat. Sweetness and sourness in meat derive from a series of amino acid and their derivatives [49]. Our results indicate that pMuSCs-NPF had comparable levels of these flavor-associated amino acids to conventional meat, suggesting a balanced flavor profile. Regarding bitterness, arginine has been identified as a key contributor [55]. Notably, pMuSCs-NPF and pMuSCs showed higher arginine concentrations than conventional meat, potentially indicating a more pronounced bitter note in cultured meat products. Collectively, our flavor-related amino acid analysis demonstrates that pMuSCs-NPF more closely approximates conventional meat in terms of aromatic, sweet, and sour characteristics. The observed umami enhancement may stem from muscle satellite cells' increased production of umami amino acids during *in vitro* culture [56]. To achieve better sensory parity with traditional meat, future cultured meat production should optimize culture conditions to mitigate bitterness. CM, as a food, was further evaluated for its digestibility. Both pMuSCs and meat underwent rapidly degradation after several hours of incubation in simulated gastric fluid at 37 °C. In contrast, the NPF scaffolds degraded more slowly due to the lack of cellulase in humans and other mammals [44]. This is corroborated by observation that the pMuSCs-NPF exhibited only a 50 % degradation rate under the same conditions (Fig. S8F). The viscous polysaccharides in NPF delay gastric emptying and slow transit through the small intestine [57]. This effect is due to the physical barrier created by the fiber, which impedes the interaction between carbohydrates and digestive enzymes, resulting in a slower degradation process. This gradual degradation positively influences gut microbiome health. Dietary fibers that are not fully digested in the upper digestive tract reach the intestines, where they serve as high-quality substrates for microbial fermentation [58]. Through microbial metabolism, short-chain fatty acids such as acetic acid, propionic acid, and butyric acid are produced. These metabolites help regulate the intestinal pH, inhibit the growth of harmful bacteria, and activate G protein-coupled receptors, thereby modulating the host's energy metabolism and immune function [59,60]. To facilitate an overall comparison among pMuSCs, NPF, pMuSCs-NPF, and meat, food properties were scored from 1 to 4 on six attributes, namely gross appearance, nutritional value, texture, juiciness, indicated flavor, and digestibility. Subsequently, a radar map was created, which demonstrated that pMuSCs-NPF exhibited a greater resemblance to conventional meat compared to pMuSCs and NPF (Fig. 7F).

Fat is an important source of essential fatty acids and lipophilic vitamins, influencing various aspects of whole-body physiology [61]. Therefore, the production of CM should aim to replicate tissue primarily composed of muscle fibers with interspersed fat. Intermuscular fats are a key determinant of meat juiciness, tenderness, and flavor. The taste panel of the 2013 cultured beef burger reported that the hamburger patty felt slightly dry due to the lack of fat [62]. Consequently, to produce CM with desired organoleptic properties, pADSCs had also been isolated and identified. Flow cytometry results confirmed positive expression of CD29 and CD44, with low expression of CD45 and CD31 in pADSCs (Fig. S9A-D) [63]. After 7 days of adipogenic induction, the Oil Red O and BODIPY staining assays demonstrated adipogenesis of pADSCs on both the NPF scaffolds and plates (Fig. 7G and Fig. S9E-G). Finally, to show a proof-of-concept for fat-containing CM on NPF scaffolds, we proposed a post-culture assembly method by stacking pMuSCs muscle blocks and pADSCs fat blocks, resulting in a pork belly prototype that reached centimeter scale dimensions (Fig. 7H). Furthermore, the vascularization of scaffold is critical for *in vitro* tissue culture, as it enables the diffusion of nutrients and oxygen, which is essential for large-scale cultured meat production. To evaluate the NPF scaffold's capacity to support cellular vascularization, we initially explored its effects on the vascularization of human umbilical vein endothelial cells (HUVECs). HUVECs were seeded onto the scaffold and induced to differentiate into vascular structures. After three days of differentiation, the cells exhibited CD31 positivity, confirming the NPF scaffold's ability to support vascularization (Fig. S10).

### 2.8 Prospects for large-scale production of pMuSCs-NPF scaffolds in CM manufacturing

**Fig. 8:**
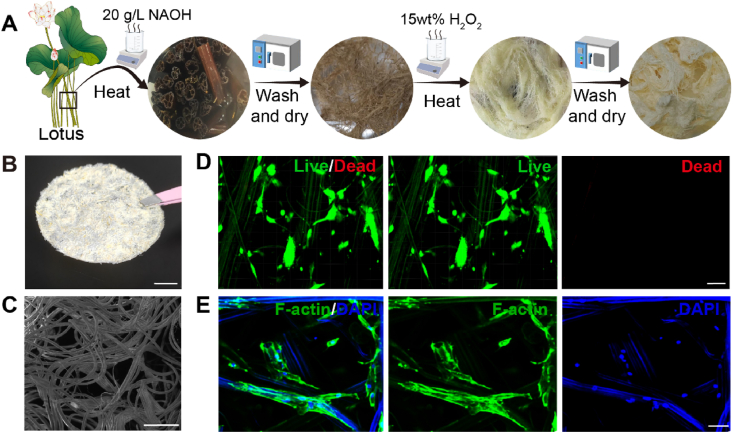
Scalable production and evaluation of NPF scaffolds for cell-cultured meat. A) Schematic overview of the process for large-scale production of NPF scaffolds. B, C) Gross morphology (B) and SEM image (C) of NPF scaffolds prepared using the scalable production method. Scale bars = 1 cm (B) and 50 μm (C). D) Live/dead cell staining of pMuSCs cultured on the scalable NPF scaffolds (green fluorescence indicates live cells and red fluorescence indicates dead cells). Scale bars = 50 μm. E) F-actin fluorescence images of pMuSCs cultured on the scalable prepared NPF scaffolds. Scale bars = 50 μm.

The industrialization of CM requires the efficient and scalable production of raw materials. Physical hand-extraction methods for obtaining lotus fiber, while effective, are labor-intensive and time-consuming, posing significant challenges for large-scale applications. To this end, we explored a modified extraction method based on previous studies [64], aiming to facilitate batch production of lotus fiber for use in CM scaffolds. The process begins with the treatment of lotus stems with NaOH, which induces a color change in the stems to brown, indicating the softening of the fibrous structure and a more pliable texture. Following multiple rinses with water, most of the pectin, waxes and water-soluble substances are removed. Further treatment with H_2_O_2_ enhances the removal of residual impurities, leading to the oxidation and subsequent whitening of the fibers. After a final washing and drying step, the fibers are obtained and can be processed into NPF scaffold (Fig. 8A and B). SEM images revealed that the structural integrity of lotus fibers extracted via this chemical method was comparable to those obtained through physical extraction, although the fibers were less orderly arranged (Fig. 8C). To address this, textile-based methods could be employed to fabricate anisotropic scaffolds suitable for further CM production [65].

To evaluate the impact of chemical extraction (using NaOH and H_2_O_2_) on scaffold edibility, we analyzed the residual of NaOH and H_2_O_2_ in the scaffolds. NaOH residues were assessed by measuring the pH of soaking solutions from both chemically and physically extracted lotus fibers using a pH meter. Results indicated no statistically significant differences in pH values between the final wash solutions of chemically and physically extracted scaffolds (Fig. S11A). For H_2_O_2_ residue analysis, we examined both the final wash solution and 24-h soaking liquid. To further assess potential digestive release, we analyzed simulated gastric fluid after 24-h incubation. H_2_O_2_ residues were undetectable in any of these samples when compared to blank controls (Fig. S11B). Structural analysis by FTIR spectroscopy showed identical spectral patterns before and after treatment, indicating no chemical modification of the native lotus fibers (Fig. S11C). Subsequently, we evaluated the biocompatibility of the scaffolds. Cytocompatibility studies demonstrated that the NPF scaffold supports cell activity without inducing cytotoxic effects (Fig. 8D). Moreover, fluorescent staining of F-actin showed that pMuSCs grew along the fibers, indicating the scaffold's potential to guide tissue organization (Fig. 8E). Taken together, the chemical method holds promise for scaling up CM production by providing a more efficient and scalable approach for lotus fiber extraction. However, making a large piece of tissue engineered meat will require vascularization and perfusion to support the survival of inner cell layers, which should be addressed in further studies.

## 3 Discussion

In this study, we present a novel method to produce CM by using lotus fiber-based NPF scaffolds. These scaffolds possess mechanical stability, structural alignment, and cytocompatibility, providing an optimal environment for the myogenic differentiation and maturation of pMuSCs. The pMuSCs-NPF composite exhibits remarkable similarities to conventional meat in terms of appearance, sensory properties, and nutritional content. Our research addresses key challenges in the field of cultured meat, particularly the development of food-grade, scalable, and sustainable scaffolds that capable of effectively mimic the intricate structure of muscle tissue, thereby advancing efficient CM production.

The selection of scaffolds is crucial in CM production, as it significantly influences cell growth, differentiation, and the overall sustainability, cost-effectiveness, and scalability of the final product. As a natural plant-based materials, lotus fiber derived from lotus stem, stands out as a highly promising material due to its exceptional renewability and sustainability [66]. Lotus is widely cultivated with a short growth cycle and strong adaptability, making it an environmentally friendly and scalable resource. As a natural polysaccharide, lotus fiber offers distinct advantages over traditional synthetic scaffold materials such as polylactic acid (PLA) and polycaprolactone (PCL). Specifically, the production of lotus fiber generates no harmful chemical waste, thereby mitigating long-term environmental impacts [67] and enhancing the sustainability of CM production. In terms of cost-effectiveness, lotus is a common agricultural crop that is relatively inexpensive and readily available. The extraction and processing of lotus fiber are straightforward and cost-efficient, requiring neither complex equipment nor energy-intensive procedures or intricate chemical reactions. This simplicity not only reduces production costs but also improves the overall cost-effectiveness of CM production. Additionally, the widespread cultivation of lotus ensures a stable and abundant supply of raw materials, which is essential for scalability. As a candidate for cell scaffolds, the hydrophilic hydroxyl groups of cellulose and specific cellulose-binding domains in lotus fiber have been shown to facilitate cell adhesion, highlighting its potential for cell culture applications without the need for additional chemical modifications or treatments. Moreover, the inherent fibrous structure of lotus fiber closely mimics the ECM of natural muscle tissue. This structural similarity makes it suitable for promoting the alignment and organization of muscle cells, facilitating the formation of myotubes and the deposition of ECM, both of which are critical for achieving meat-like texture.

The superior mechanical properties of scaffolds are critically important for the *in vitro* reconstruction of muscle tissue. We noticed that the Young's modulus of lotus fiber is higher than that of *in-vivo* muscle tissues [68]. This discrepancy in mechanical strength could have several implications for the final cultured meat product. For instance, it may lead to a denser or firmer texture in the cultured meat compared to conventional meat, potentially affecting its sensory qualities. Additionally, the rigidity of the scaffold might influence nutrient transport and waste removal within the cultured meat construct, thereby impacting cell viability and tissue development. To address these potential challenges, further research is needed to fine-tune the mechanical properties of the scaffold. One promising approach is enzymatic pretreatment, which can adjust the mechanical properties of lotus fiber to better match the characteristics of animal tissues, as has been explored in relevant studies [69]. This adjustment would enhance the suitability of lotus fiber for cultured meat production, ensuring its effective application in this field.

To gain a comprehensive understanding of the pMuSCs-NPF composite, our study extended beyond the analysis of cell morphology and a limited set of biomarkers. We employed transcriptomic and proteomic analyses to provide deeper insights into the cellular and molecular dynamics. These analyses revealed significant upregulation of myogenic differentiation markers and enhanced ECM production. The ECM is acknowledged as a critical mediator of biophysical signals and a regulator of cellular behaviors, including adhesion, proliferation, migration, and differentiation [70]. The increased ECM secretion by pMuSCs-NPF suggests its potential to modulate ECM organization, thereby influencing pMuSCs behaviors and promoting muscle development. Notably, fibronectin, a key ECM protein that provides a structural scaffold for myoblasts adhesion and alignment, facilitating their fusion into multinucleated myotubes. In the pMuSCs-NPF composite, fibronectin expression was markedly augmented. Its dynamic presence in the ECM ensures proper tissue organization and function during muscle development [71]. In addition, CDH11 and CDH13, members of the cadherin family of cell adhesion molecules, play crucial roles in both the early stages of muscle cell commitment and the later stages of differentiation and tissue formation [46]. Their expression levels were also notably elevated, which is essential to ensure the correct development and function of muscle tissue. Moreover, the synergistic interaction between fibronectin and CDH11/13 is particularly important in contexts such as musculoskeletal development and tissue repair. Although it remains unclear which specific components plays the most critical role in the differentiation and maturation of pMuSCs-NPF, this uncertainty represents an important area for further investigation.

Conventional methods for increasing the volume of CM volume often rely on aggregating microtissues or employing 3D printing techniques with crosslinking agents [[72], [73], [74]] However, these approaches may compromise taste or pose potential risks to human health. In contrast, adjusting the NPF scaffold size offers a potential strategy for scaling up CM production from micro- or millimeter-scale to centimeter-scales, bringing the production process closer to achieving the dimensions of real meat. Apart from mimicking muscle tissue, our study also replicates the arrangement of muscle and fat to emulate the composition of real meat. While there existing literature on varying meat component proportions [72,73,75,76], our proposed scaffolds facilitate the manipulation of meat component ratios, underscoring the innovative nature of our methodology in allowing customizable adjustments to muscle and fat ratios. For whole-cut meat, co-culture of pMuSCs and pADSCs presents a viable strategy to replicate the intricate fat marbling characteristic of animal-based meat, enhancing the texture, structure, and flavor of CM, and potentially improving its marketability. While this study has demonstrated that the NPF scaffold can support the vascular differentiation of HUVECs, the construction of a composite scaffold with a functional vascular network is essential for advancing the industrial application of cultured meat technology. Future studies should focus on integrating co-culture systems, growth factor modification, and microfluidic technologies to further investigate the vascularization potential of the NPF scaffold. Specifically, co-culturing porcine endothelial cells with pMuSCs will be beneficial for large-scale production of CM.

Finally, it is important to note that our current system utilizes serum-based media containing adherent proteins, which promote cell adhesion. For future research, it is essential to explore the use of a serum-free culture system, such as the serum-free media formulations previously described [[77], [78], [79]]. This can also be achieved by modifying the lotus fiber with additional nutrients or bioactive compounds, which will help to promote cell adhesion and differentiation under serum-free conditions, thereby enabling more efficient CM production.

## 4 Conclusion

This study introduces a pioneering approach to CM production by developing a novel lotus fiber-based NPF scaffold combined with pMuSCs. This scaffold demonstrates mechanical stability and excellent cytocompatibility, closely mimicking the structural environment of natural muscle tissue. This similarity facilitates the myogenic differentiation and maturation of pMuSCs, enabling the formation of organized muscle-like structures. The resulting pMuSCs-NPF exhibits comparable appearance, organoleptic characteristics, and nutritional value to conventional meat, offering significant potential as a bottom-up strategy for CM construction. Furthermore, the scalability of the lotus fiber extraction method enhances the feasibility of this approach for large-scale, sustainable meat production. These findings establish a robust foundation for advancing sustainable, ethical, and nutrient-rich meat alternatives to address the growing demand for protein in a resource-efficient manner.

## 5 Experimental section

### 5.1 Preparation of NPF scaffolds

Lotus stalks were harvested from the lotus root fields in the Yuelu region of Changsha, Hunan province. We prepared the NPF scaffolds through a physical method. Briefly, lotus stems were thoroughly cleaned with a gentle flow of pure water before stretching the fibers. The lotus petioles were gently sliced with a knife and pulled in opposite directions from both ends to extract the lotus fiber. The extracted fibers were then aligned and uniformly wrapped around a rotating drum to achieve a consistent thickness of 150 ± 20 μm for each individual one. The resultant fiber mats were carefully detached from the drum for further use.

The large-scale extraction method for lotus fiber was modified according to a previous study [64]. Briefly, dried lotus stems were immersed in 20 g/L NaOH (Sinopharm Chemical Reagent, 10019718) in a boiled water bath for 30 min. The lotus stems were then washed in deionized water to neutralize the pH and dried at 50 °C. Subsequently, the dried samples were rinsed in 15 wt% H_2_O_2_ (Sinopharm Chemical Reagent, 80070961) in a boiled water bath for 15 min. Finally, the lotus fibers were rinsed for neutralization and dried at 50 °C. For cell culture, the scaffolds were sterilized with 75 % ethanol (Sinopharm Chemical Reagent, 80176961) for 4 h, washed at least three times in PBS (Servicebio, G4202), and exposed to UV radiation overnight.

### 5.2 Scanning electron microscopy (SEM) analysis

The morphology of NPF scaffold was observed using SEM (MIRA3, TESCAN, Czech Republic) at an acceleration voltage of 15 kV. The cross-sectional image was obtained by cutting the sample in liquid nitrogen, and all water samples were air dried. Samples were then mounted on aluminum stubs with the assistance of carbon tape, and sputter-coated with gold before observation. Fiber fineness was measured using ImageJ software (v1.8.0, NIH, USA).

### 5.3 Measurement of water contact angle

To quantify wettability and stability of the NPF scaffold, the static water contact angle measurement was performed by using a sessile drop method (SDC-200, SinDin, China). In brief, a 5 μL droplet of deionized water was deposited onto the scaffold surface, and the initial contact angle (0 s) was recorded from dynamic image analysis.

### 5.4 Zeta potential analysis

Lotus fibers were washed to remove surface contaminants, sectioned into small fragments, and dispersed in deionized water. The suspension was ultrasonicated for 30 min, filtered through filter paper, and analyzed using a zeta potential analyzer (Zetasizer Nano ZS, Malvern Panalytical, UK).

### 5.5 Fourier transform infrared spectroscopy (FTIR) analysis

The functional groups present in the lotus fiber were identified using a FTIR instrument (Nicolet iN10, Thermo, USA). The scaffold was crushed into a fine powder and compressed into tablets using KBr. The infrared spectra were obtained at room temperature across a range of wavenumbers from 650 to 4000 cm^−1^.

### 5.6 Tensile test

The mechanical properties of NPF scaffolds were measured using a mechanical testing instrument (HYC-2011, Hong Jina, China) equipped with a 0.02 N load cell. The stress-strain curves of NPF were generated by subjecting the material to a constant longitudinal stretching rate of 20 mm/min until breakage. Young's modulus was derived from the stress-strain curves, and the fracture stress were determined from the peak of the curve.

### 5.7 Cell isolation and culture

One-week-old large white piglets were purchased from Jiahe Agriculture and Animal Husbandry Co., Ltd. ecological agriculture division. The experimental piglets were performed following the rules of ethical animal treatment. pMuSCs were isolated according to a previous published method [29]. Briefly, porcine peroneus tertius muscle tissues were freshly collected, washed with cold PBS containing 1 % (*v/v*) penicillin-streptomycin (P/S, Gibco, 15070063), and excess tissues were removed. The muscle tissues were then mechanically dissected and enzymatically dissociated using 1 mg/mL pronase (Roche, 10165921001) and 1 mg/mL dispase II (Roche, 04942078001) at 37 °C for 1.5 h. Throughout the procedure, the mixture was agitated using a pipette at 10-min intervals until a smooth, fluid consistency was achieved. The resulting cell suspension was then filtered through 100 μm and 70 μm cell strainers sequentially, followed by centrifugation at 800*g* for 10 min. The pelleted cells were resuspended in growth medium consisting of 1640 medium (Gibco, C11875500BT) supplemented with 20 % (*v/v*) fetal bovine serum (FBS, Gibco, S-FBS-SA-015), 5 ng/mL basic fibroblast growth factor (bFGF, Sino Biological Inc, 10014-HNAE), 1 % (*v/v*) non-essential amino acids (Gibco, 11140-050), 0.5 % (*v/v*) chicken embryo extract (GEMINI, 100-163P), 1 % (*v/v*) GlutaMax (Gibco, 35050), and 1 % (*v/v*) P/S. To remove fibroblast, the cell suspension was initially seeded on plates coated with 0.05 % (*v/v*) collagen I (Corning, 354236) for 1 h. Subsequently, individual cells that unattached were transferred to a new collagen I-coated plate at a density of 5 × 10^5^ cells/cm^2^ for subculture. Upon reaching 60 % confluency, pMuSCs were trypsinized for further passaging or cryopreservation. To confirm the myogenic capacity, cells from passages 1 to 4 were used in the subsequent experiments.

pADSCs were obtained following established protocols [80]. Briefly, the adipose tissues were collected from subcutaneous regions, washed in cold PBS containing 1 % (*v/v*) P/S. After mechanical mincing, the tissues were subjected to digestion using 0.1 % (*w/v*) collagenase I (Sigma, C0310) at 37 °C for 3 h, and then terminated by the addition of growth medium (DMEM, Gibco, C11885500BT) supplied with 10 % (*v/v*) FBS and 1 % (*v/v*) P/S). After digestion, the resultant mixture was sequentially filtered with 100 μm and 70 μm cell strainers, and centrifugation at 1000*g* for 10 min. Finally, cells were resuspended in growth medium for subculture. For routine expansion, the medium was refreshed every three days.

### 5.8 Cell differentiation

Sterilized NPF scaffolds were immersed in growth medium before cell seeding. For myogenic differentiation, pMuSCs were induced when reaching 90 % confluence using a differentiation medium consisting of DMEM (Gibco, C11995500BT) supplemented with 0.4 % (*v/v*) Ultroser G (Pall, 15950-017) and 1 % (*v/v*) P/S. The cells underwent differentiation for one week, with the medium being refreshed every 2–3 days.

For adipogenic differentiation, pADSCs were seeded on the scaffold at a density of 7 × 10^4^ cells/cm^2^ and incubated at 37 °C for 2 h to allow cell adhesion. Subsequently, the induction medium composed of DMEM, supplemented with 10 % (*v/v*) FBS, 5 μg/mL insulin (Gibco, 51500056), 1 μM dexamethasone (Sigma, D4902), and 0.5 mM isobutyl methylxanthine (Sigma, I5879) was added. The cells underwent a two-week differentiation process, with the medium being refreshed every 2–3 days.

### 5.9 Immunofluorescence staining

Specimens were fixed in a 4 % (*w/v*) paraformaldehyde solution (Servicebio, G1101) for 20 min and permeabilized with 0.1 % Triton X-100 (Beyotime, ST1723) in PBS for 10 min at room temperature. After blocking with 5 % goat serum in PBS for 1 h, cells were incubated with diluted primary antibodies overnight at 4 °C. The primary antibodies used in this study include PAX7 (Developmental Studies Hybridoma Bank, AB528428), MYOD (Proteintech, 66214), MyHC (Bio-Techne, MF20), and Desmin (Santa Cruz, sc-23879). After washing with PBS, the cells were incubated with Alexa Fluor 488 goat anti-mouse (Invitrogen, A-11001) and Alexa Fluor 546 goat anti-rabbit antibodies (Invitrogen, A-11035) for 1 h at room temperature. Cell nuclei were stained with DAPI (Beyotime, C1005) for 10 min, and images were captured using a confocal fluorescence microscope (STELLARIS 5, Leica, Germany). ImageJ software was used for quantitative analysis, with six randomly selected fields of view were analyzed from the stained samples. PAX7-positive cells were identified based on GFP signal, while the total number of cells was determined using DAPI signal. The percentage of PAX7-positive cells per field was calculated by dividing the number of PAX7-positive cells by the total number of cells. The final percentage represents the mean value across all six fields. For the assessment of muscle maturity, the fusion index was calculated by quantifying the proportion of nuclei present in multinucleate myotubes with more than two nuclei. The maturation index was calculated by determining the proportion of myotubes with more than five nuclei relative to the total number of myotubes [81].

### 5.10 Fluorescence-activated cell sorting (FACS) analysis

pMuSCs and pADSCs were characterized for the presence of typical surface markers of muscle stem cell or adipose stem cell using flow cytometry. Briefly, after a three-day culture, pMuSCs suspensions were stained with a mixture of FITC-conjugated anti-CD29 (BioLegend, 303015) and PE-conjugated anti-CD56 (BioLegend, 304605) antibodies for 20 min pADSCs were stained with 1 μg PE- or FITC-conjugated antibodies, including CD29-FITC, CD44-FITC (eBioscience, 11-0441-81), CD45-PE (BioLegend, 982322), and CD31-FITC (BioLegend, 989002) for 20 min, respectively. All flow cytometry antibodies were obtained from BioLegend. Unstained pMuSCs or pADSCs were served as the negative control. The samples were analyzed by flow cytometry (CytoFLEX, Beckman, China), and data were processed using FlowJo software.

### 5.11 Evaluation of cell viability, proliferation, and morphology

Cell viability was conducted using a live/dead cell viability kit (Beyotime, C2015M). After 3 days of cultivation on NPF scaffolds or plates, cells were stained with Calcein (live cells) and PI (dead cells) according to the manufacturer's guidelines. Subsequently, the cells were visualized utilizing a confocal fluorescence microscope.

Cell proliferation assay was performed utilizing the Cell Counting Kit-8 (Bimake, B34304). Cells were initially seeded on NPF scaffolds or plates at a density of 2 × 10^3^ cells/cm^2^. The cell density was then evaluated on day 1, 3, 5, and 7 using a 10 % CCK-8 solution in a 5 % CO_2_ incubator at 37 °C for 3 h, after which the absorbance of the medium was measured at 450 nm with a microplate absorbance spectrophotometer (PerkinElmer).

The morphology of pMuSCs cultured on the NPF scaffolds or plates was visualized by fluorescent staining with F-actin (Thermo, A12379). After 4 days of cultivation on either NPF scaffolds or uncoated culture plates, the cells were fixed with 4 % (*w/v*) paraformaldehyde for 20 min at room temperature. Subsequently, the cytoskeletons of cells were stained with F-actin, and cell nuclei were stained with DAPI. Fluorescent images were captured using a confocal fluorescence microscope. Cell orientation was calculated using the directionality plug-in in ImageJ software.

### 5.12 DiI staining

To visualize cell filtration and spreading in NPF scaffolds, pMuSCs were pre-stained with DiI (Beyotime, C1991S). Briefly, pMuSCs were incubated with 5 μg/mL of DiI for 20 min at 37 °C, after which the solution was aspirated and washed with PBS for at least three times. Subsequently, the cells were seeded onto the NPF scaffolds. 3D reconstruction scans were performed, and images were captured using a confocal fluorescence microscope.

### 5.13 Quantitative RT-PCR

The total RNA was extracted using Trizol (Invitrogen, 15596018CN), and RNA was then reverse-transcript to cDNA using PrimeScript™ RT Master Mix (Takara, RR036A) in accordance with the manufacturer's instructions. RT-qPCR was performed in triplicate with TB Green premix (Takara, RR820A) on a Light Cycler apparatus (CFX96TOUCH, Bio-Rad, USA). The primers utilized in the study are presented in Table S1. The relative expression levels of each target gene were calculated using the 2^−ΔΔCT^ method. All experiments included at least three biological replicates, and representative results are shown as target gene normalized to GAPDH.

### 5.14 Western blots analysis

The proteins of cells cultured on NPF scaffolds or plates were extracted using RIPA lysis buffer (Epizyme, PC101) supplemented with protease inhibitor cocktail (Beyotime, P1050) and phenylmethanesulfonylfluoride (Beyotime, ST507). Protein content was quantified using the bicinchoninic acid assay (BCA) kit (Epizyme, ZJ101). The protein lysates were separated by 10 % SDS-PAGE (Epizyme, PJ112) and transferred onto a polyvinylidene difluoride membrane (Millipore, IPVH00010). Primary antibodies targeting Desmin, Fibronectin (Santa Cruz, sc-73611), MyHC, Collagen I (Bioss, bs-7158R), and GAPDH (Beyotime, AF0006) were incubated overnight at 4 °C. The resulting blots were then incubated with HRP-conjugated secondary antibodies (abiowell, AWS0001a or AWS0002a) for 1.5 h. Protein bands were detected using a horseradish peroxidase chemiluminescence detection kit (Thermo, 34075) and imaged with the Image Quant LAS 4000 system. The quantification of protein levels was conducted using ImageJ software.

### 5.15 Transcriptome analysis

#### 5.15.1 Library preparation

Total RNA was isolated using the Trizol Reagent. RNA concentration, purity, and integrity were assessed using a NanoDrop spectrophotometer (Thermo, USA). Purified library fragments were enriched with the AMPure XP system (Beckman, USA). DNA fragments with ligated adaptor molecules on both ends were selectively enriched using Illumina PCR primer cocktail in a 15 cycle PCR reaction. Purification of products was carried out using the AMPure XP system, and quantification was performed using the Agilent high-sensitivity DNA assay on a Bioanalyzer 2100 system (Agilent, USA). The sequencing library was then subjected to sequencing on the NovaSeq 6000 platform (Illumina, USA) with pair-end 150 bp (PE150) reads performed by Personalbio Company (Shanghai)

#### 5.15.2 Reads mapping and gene expression quantification

After the cDNA library samples underwent sequencing as per the aforementioned methodology. Reads containing adapters, those with 'n' nucleotides exceeding 10 %, and low-quality reads with a sequencing quality score of 5 or lower were eliminated. The remaining reads underwent quality assessment using FastQC (v.0.11.5). Clean reads were mapped to the Sscrofa11.1 reference genome using STAR (v.2.5.3a). Subsequently, the raw counts matrix was generated using feature counts. Gene expression quantification was normalized using counts per million reads (CPM), and further filtering was based on the sum of relative abundance.

#### 5.15.3 Identification of DEGs and functional enrichment analysis

DEGs in pairwise comparisons were identified using the R package edgeR, with genes showing at least a two-fold change in expression and FDR <0.05 considered significant. KEGG pathway analysis was conducted using the R package clusterProfiler (v4.6.0). GSEA (v4.1.0) was employed to assess enrichment across all genes.

### 5.16 Label free relative quantitative proteomic analysis

#### 5.16.1 Protein extraction and digestion

After 7 days of differentiation, pMuSCs cultured on NPF scaffolds or plates were washed three times with PBS. Loin meat from adult large white pigs was collected within 30 min of slaughter and immediately frozen with liquid nitrogen. All samples were transferred on dry ice to Personalbio Company (Shanghai) for further analysis. Sample lysis and protein extraction were performed using SDT buffer (4 % SDS, 100 mM Tris-HCl, 1 mM DTT, pH 7.6). Peptides in each sample were digested and desalted using C18 Cartridges (Empore™ SPE Cartridges C18, standard density, Sigma, USA). After desalting, the peptides were concentrated via vacuum centrifugation and reconstituted in a solution containing 40 μL of 0.1 % (*v/v*) formic acid.

#### 5.16.2 LC-MS/MS analysis and date processing

The Q Exactive Mass Spectrometer (Thermo, USA) was utilized for LC-MS/MS analysis. Peptides were loaded onto a reverse phase trap column (Thermo, USA) connected to the C18-reversed phase analytical column (Thermo, USA) using buffer A (0.1 % formic acid) and separated with a linear gradient of buffer B (84 % acetonitrile and 0.1 % formic acid). The mass spectrometer operated in a positive-ion mode with peptide recognition functionality activated. The raw data from each sample were aggregated and subsequently analyzed using the MaxQuant software for the purpose of identification and quantitation analysis.

#### 5.16.3 Identification of differentially expressed proteins (DEPs) obtain and functional enrichment analysis

DEPs in pairwise comparisons were identified using the R package edgeR. Proteins with at least a two-fold change in expression and FDR <0.05 were considered differential expressed. A *k*-means approach was used (R package pheatmap) to cluster all the DEPs identified through pairwise comparisons. The R package clusterProfiler (v4.6.0) was used to perform KEGG pathway enrichment analysis of DEPs (*p* < 0.05). Additionally, annotations were plotted by R scripts. GSEA (v4.1.0) was employed to conduct an enrichment study on all proteins.

### 5.17 Untargeted metabolomic determination and analysis

Samples were individually ground using liquid nitrogen, and the resulting homogenate was resuspended by vigorous vortexing with prechilled 80 % methanol. After sonication in an ice water bath for 6 min, the samples were incubated on ice for 5 min, followed by centrifugation at 4 °C for 20 min. Subsequently, the supernatant was injected into the liquid chromatography-tandem mass spectrometry (LC-MS/MS) system provided by Novogene Company (Beijing) for analysis. The raw data files were processed using the Compound Discoverer 3.3 (CD3.3, Thermo) to perform peak alignment, peak picking, and quantitation for each metabolite. Detected peaks were then matched with the mzCloud (https://www.mzcloud.org/),mz Vault, and Mass List database to obtain accurate qualitative and relative quantitative results. For non-normally distributed datasets, standardization was performed using the formula, sample raw quantitation value/(the sum of sample metabolite quantitation value/the sum of QC1 sample metabolite quantitation value) to obtain relative peak areas. Compounds with coefficient of variation (CV) values exceeding 30 % in quality control (QC) samples were excluded. Consequently, the identification and relative quantification results of the metabolites were obtained.

### 5.18 Texture profile analysis (TPA)

The textural properties of all samples were tested using a texture analyzer (TA1, Lloyd Instruments, UK) at 25 °C. The experimental parameters were as follows: All samples were prepared with dimensions of 2 cm in length, 2 cm in width, and 0.5 cm in thickness. Compression was conducted using a TMS 12.7 mm Black Aoetate (AOAC 12.7 mm) probe at a pre-test, test, and post-test speeds of 2 mm/s, 1 mm/s, and 2 mm/s, respectively. The compression reached a 75 % compression ratio with a trigger force of 20 g. The probe repositioned itself to its initial activation position and paused for 5 s before commencing the next cycle. The following texture parameters were measured: hardness (peak force observed during the initial compression cycle), springiness (vertical displacement a material undergoes to regain its original height, measured from the completion of the first bite to the initiation of the second bite), chewiness (calculated as the product of hardness, cohesiveness, and springiness), and gumminess (refers to the amount of energy needed to masticate semi-solid food until it reaches a suitable consistency for swallowing, expressed numerically as the product of hardness and cohesiveness). All parameters were calculated by the NexyGen Lloyd software version 4.1 (Lloyd Instruments Ltd).

### 5.19 Rheology analysis

For rheology analysis, pMuSCs, NPF, pMuSCs-NPF, and meat were prepared as cylindrical disks with a thickness of 1 mm and a diameter of 1 cm. These samples were then tested using a rheometer (MCR92, Anton Paar, Austria). All samples underwent compression at angular frequencies from 100 to 0.1 rad/s, and the storage modulus (G′) and loss (G″) modulus were measured and recorded.

### 5.20 Analysis of protein content

The protein contents of pMuSCs, NPF, pMuSCs-NPF, and meat were assessed using a BCA detection method. Samples were lysed with RIPA lysis buffer at 4 °C for 30 min. After centrifugation at a speed of 10,000×*g* for 10 min, proteins were extracted from the supernatant. Protein quantification was performed as described in the Western blot procedure.

### 5.21 Assessment of total collagen content

The total collagen content in CM samples was quantified utilizing the hydroxyproline assay kit (Jiancheng Bioengineering Institute, A030-2-1), following the manufacturer's guidelines. The absorbance of the resultant complex was subsequently measured at a wavelength of 550 nm. The total collagen content was calculated using a standard curve generated with known concentrations of hydroxyproline. The calculation was applied using the following formula:

(1) $$Total\;collagen\;\left(\mu g/ml\right)=\frac{A_{sample}-A_{blank}}{A_{s\;\tan\;dard}-A_{blank}}\times C_{s\;\tan\;dard}\times N$$

*C*_*standard*_ is the concentration of standard sample (5 μg/mL), and *N* is the dilution ratio of the tested samples.

### 5.22 Moisture and cooking loss

The moisture content and cooking loss of all samples were assessed using the following procedure: First, the raw weight of the samples was measured after removing excess solution with a Kimwipe (Kimberly-Clark, 34155). Next, the lyophilized weight was determined by freeze-drying the samples for two days. The cooked weight was then calculated by cooking the samples on an electric hot plate at 120 °C for 3 min. Subsequently, the cooked samples were cooled to 25 °C and weighed again. The moisture content and cooking loss were determined using the following formulas:

(2) $$Moisture\;content\;\left(\%\right)=\frac{Raw\;weight\left(g\right)-Lyophilized\;weight\left(g\right)}{Raw\;weight\left(g\right)}\times 100\%$$

(3) $$Cooking\;Loss\;\left(\%\right)=\frac{Raw\;weight\left(g\right)-Cooked\;weight\left(g\right)}{Raw\;weight\left(g\right)}\times 100\%$$

### 5.23 *In vitro* digestion test

The *in vitro* digestion experiment was carried out following a previously published method [82]. Briefly, samples were immersed in simulated gastric fluid (0.2 % (*w/v*) NaCl, and 2 mg/mL pepsin in 0.7 % (*v/v*) HCl), and placed in an incubator at 37 °C with 5 % CO_2_. Excess solution was gently removed from the sample, the remaining weight of the samples at indicated time points was recorded and represented as a percentage.

### 5.24 Assessment of product properties

To thoroughly evaluate the CM product, a comprehensive evaluation was conducted based on six key attributes including gross appearance, nutritional value, texture, juiciness, indicated flavor, and digestibility of pMuSCs, NPF, pMuSCs-NPF, and traditional meat. For gross morphology analysis, samples from all four groups were standardized to uniform dimensions. 30 participants were recruited for this test. Pure pMuSCs were prepared by harvesting cells from culture plates using a cell scraper and stacking them together. Subsequently, pMuSCs, NPF, and pMuSCs-NPF samples were stained with 0.5 % (*w/v*) heme (Beyotime, ST1375) to enhance visual comparison. A scoring system was employed to quantify the similarity of each attribute to conventional meat, with a score of 4 representing the highest degree of similarity to conventional meat. The detailed scoring criteria are provided in Table S2. A radar map was drawn to graphically visualize the sensory evaluation scores.

### 5.25 Detection of NaOH and H_2_O_2_

For NaOH detection, a pH meter (STARTER3100, OHAUS, USA) was employed. The pH meter was calibrated according to the manufacturer's instructions. After calibration, the electrode was rinsed with distilled water and dried with filter paper to remove any residual moisture. The electrode was then inserted into the solution to be tested, which included the final wash solution of the scaffold treated with NaOH and H_2_O_2_, the liquid after soaking for one day, and the liquid from the untreated scaffold. The pH value of the solution was recorded once the reading stabilized.

The concentration of H_2_O_2_ was determined using a detection kit (Solarbio, BC3590) according to the manufacturer's guidelines. The samples included the final wash solution of the scaffold treated with NaOH and H_2_O_2_, the liquid after soaking for one day, and the samples soaked in simulated gastric fluid for one day. A spectrophotometer (UV-1900i, SHIMADZU, Japan) was used to measure the absorbance of the test samples at a wavelength of 415 nm.

### 5.26 Oil Red O and BODIPY staining

After three weeks of adipogenic differentiation, samples were washed three time with PBS, and then fixed with 4 % (*w/v*) paraformaldehyde for 30 min at room temperature. After that, samples were incubated with a 1 % (*w/v*) filtered Oil Red O (Beyotime, C0157S) solution or BODIPY (Beyotime, C2053S) for 15 min. Subsequently, stained lipid droplets within the cells were examined and captured using an inverted phase contrast microscope (TE2000, Nikon, Japan).

### 5.27 Stack-assembly of centimeter-scale pork belly

After 7 days of differentiation, muscle blocks from pMuSCs and fat blocks from pADSCs were collected and washed with PBS. To recreate the color of fresh meat, muscle blocks were stained with heme. Based on the contour of pork belly, muscle and fat blocks were meticulously stacked to a size at the centimeter scale to replicate the natural arrangement.

### 5.28 Angiogenic differentiation of HUVECs on scaffolds

To induce angiogenesis of HUVECs, the cells were seeded onto the scaffold at a density of 1 × 10^5^ cells/cm^2^. The seeded cells were then incubated at 37 °C in a humidified atmosphere containing 5 % CO_2_ for 3 h to ensure proper cell adhesion. Subsequently, the induction medium was added, which consisted of DMEM supplemented with 5 % (*v/v*) FBS, 10 ng/mL (*w/v*) vascular endothelial growth factor (VEGF, PeproTech, 100-20), and 1 % (*v/v*) P/S. The cells were cultured for 3 days to allow for angiogenic differentiation, with the medium being refreshed every 2 days.

### 5.29 Statistical analysis

All data were presented as the mean ± SD. The statistical analysis was performed with GraphPad Prism 8. The *t*-test was employed for comparing the two treatment groups. For the analysis of more than two groups, a One-way ANOVA was conducted, followed by Bonferroni's multiple comparison tests. The threshold for statistical significance was established at a level of *p* < 0.05.

## CRediT authorship contribution statement

**Yueren Wu:** Writing – original draft, Methodology, Data curation, Investigation. **Yajun Li:** Data curation, Investigation. **Qing Yang:** Data curation, Investigation. **Chaoyong He:** Investigation, Methodology. **Jianping Tang:** Conceptualization. **Liyang Shi:** Supervision, Conceptualization. **Jianwu Dai:** Funding acquisition, Supervision, Conceptualization. **Can Zhang:** Funding acquisition, Supervision, Writing – review & editing, Conceptualization.

## Ethics approval and consent to participate

This research does not involve experimentation on animals.

## Declaration of competing interest

The authors declare the following personal relationships which may be considered as potential competing interests: Jianping Tang is currently employed by Chenxi Xinchuang Biological Technology Co., Ltd.
