# Supplementary material for: Lotus fiber-derived scaffolds for enhanced cultured meat production: Quality and sustainability
Source: Bioact Mater. 2025 Jun 27;51:807–24. doi: 10.1016/j.bioactmat.2025.06.048 (PMC12268086; doi:10.1016/j.bioactmat.2025.06.048)
Supplement: Multimedia component 1 [file mmc1.pdf]

Supplementary Materials for

Lotus Fiber-Derived Scaffolds for Enhanced Cultured Meat Production: Quality and Sustainability

This file includes:

Figs. S1 to S11

Tables S1 to S2

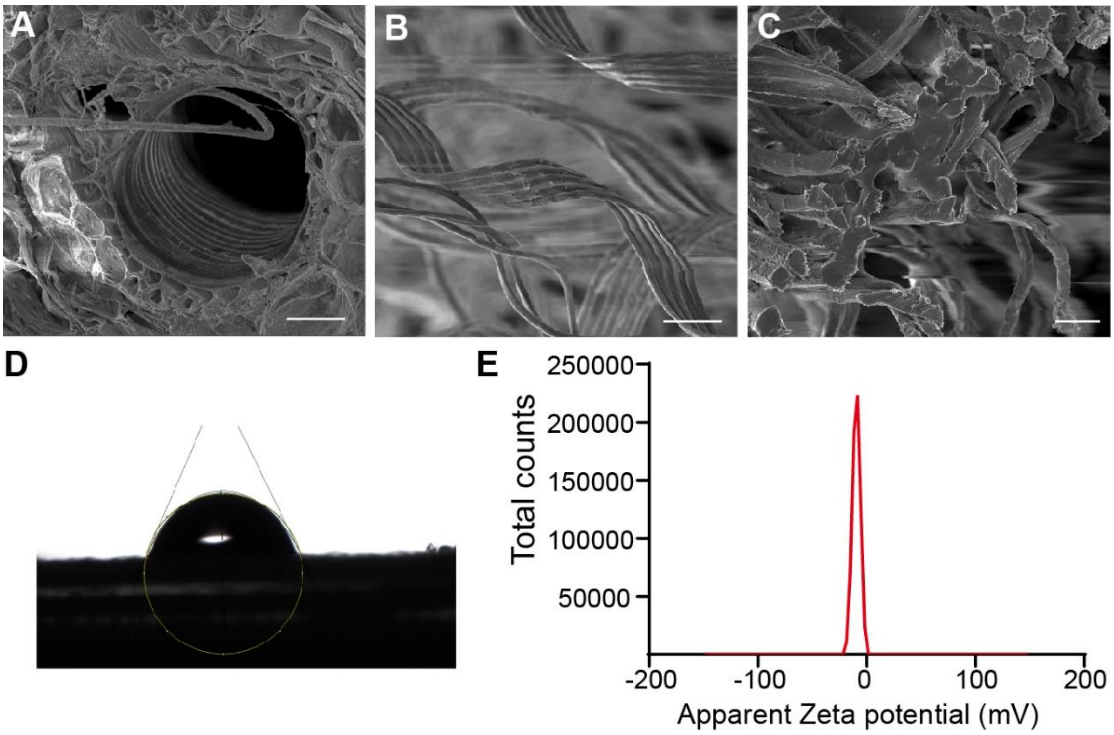

Fig. S1. Characterization of the NPF scaffold. A, B) SEM image of the cross section of a tracheary element (A), single spiral lotus fiber bundle (B), and the cross-section of the NPF scaffold (C). Scale bars = 50 μm (A, B) and 10 μm (C). D) Water contact angle images of the NPF scaffold. E) Zeta potential measurement of the NPF scaffold (n=3).

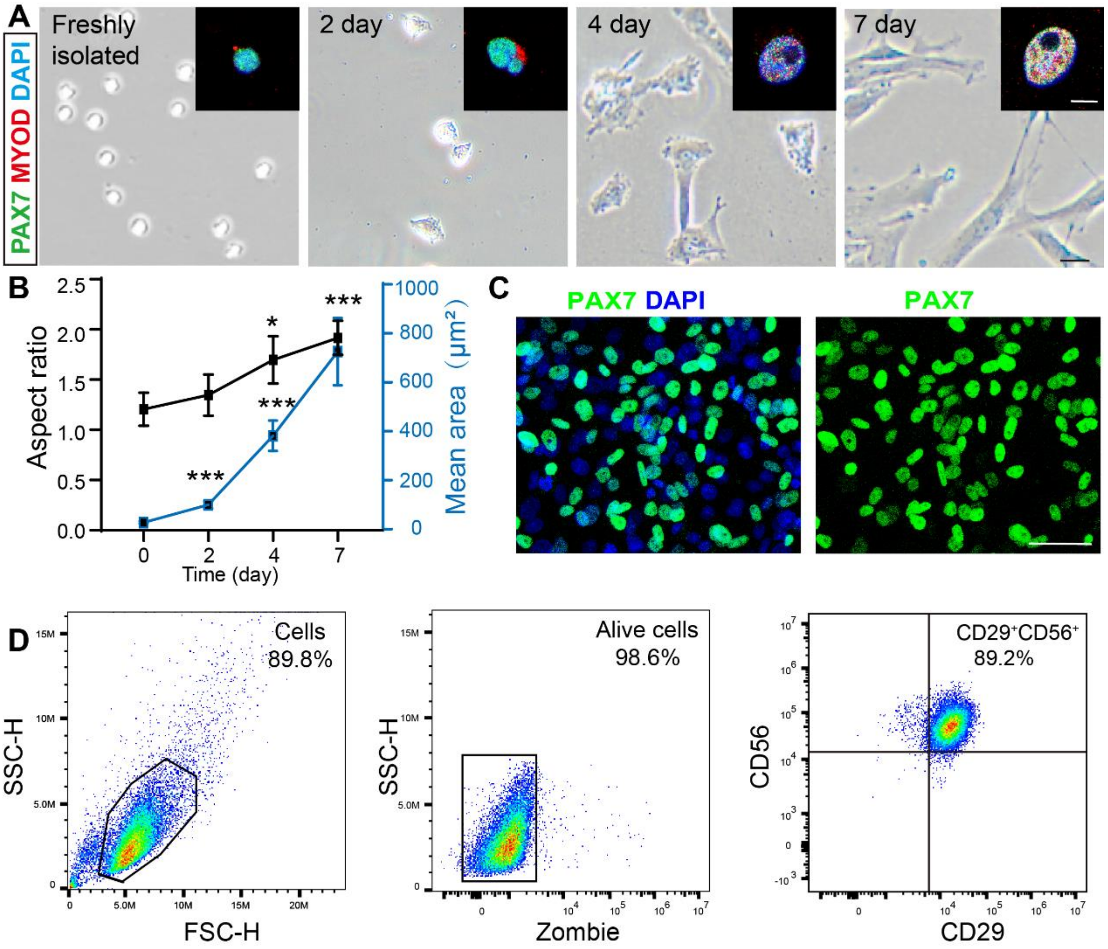

Fig. S2. Isolation and identification of pMuSCs. A) Representative phase contrast images of pMuSCs at different time points post-isolation. Scale bars = 10 μm. The insert shows the expression of PAX7 and MYOD in pMuSCs at indicated days. Scale bars = 5 μm. B) Quantification of the aspect ratio and mean area of pMuSCs over a period of 7 days in culture (n = 15). C) Immunofluorescent staining of PAX7 in pMuSCs cultured 3 days *in vitro*. Scale bars = 50 μm. D) Flow cytometry analysis of muscle stem cells markers CD29 and CD56 expression. Results are presented as mean ± SD, statistical comparison was performed by a two-tailed t test. * $p < 0.05$, *** $p < 0.001$.

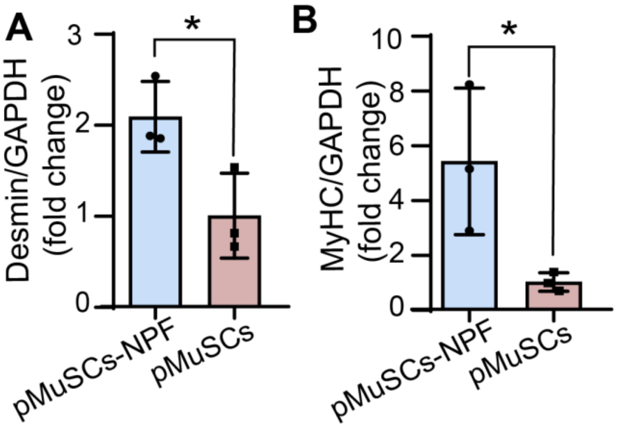

Fig. S3. Myogenic differentiation of pMuSCs on NPF scaffold and plate. A, B) Quantification analysis of Desmin and MyHC protein expression levels, normalized to GAPDH, in pMuSCs-NPF and pMuSCs groups (n = 3). Results are presented as mean ± SD, statistical comparison was performed by a two-tailed t test. * $p < 0.05$.

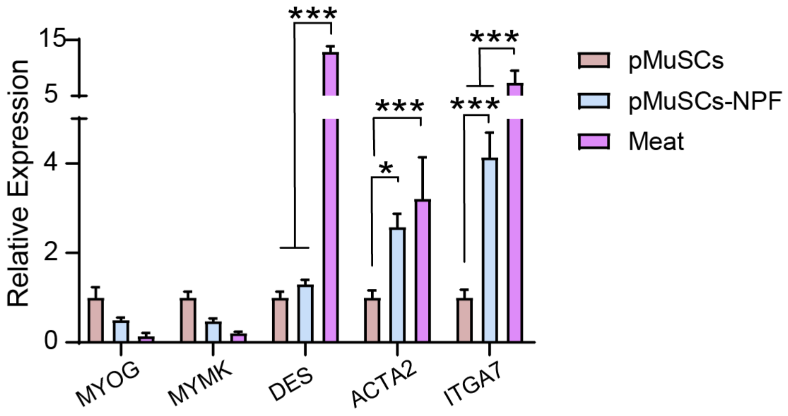

Fig. S4. RT-qPCR analysis of MYOG, MYMK, DES, ACTA2, and ITGA7 in pMuSCs, pMuSCs-NPF, and meat groups. The fold change is normalized to the pMuSCs group (n = 3).

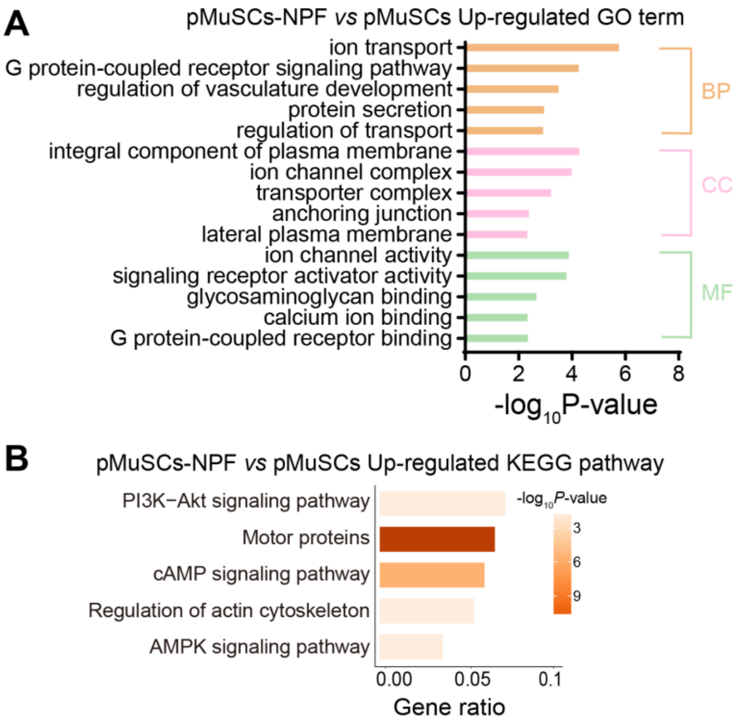

Fig. S5. Comprehensive transcriptome analysis between pMuSCs-NPF and pMuSCs. A) Representative up-regulated GO term in pMuSCs-NPF compared to pMuSCs. The terms are categorized into three main groups: Biological Process (BP), Cellular Component (CC), and Molecular Function (MF). B) Representative up-regulated KEGG pathway in pMuSCs-NPF relative to pMuSCs.

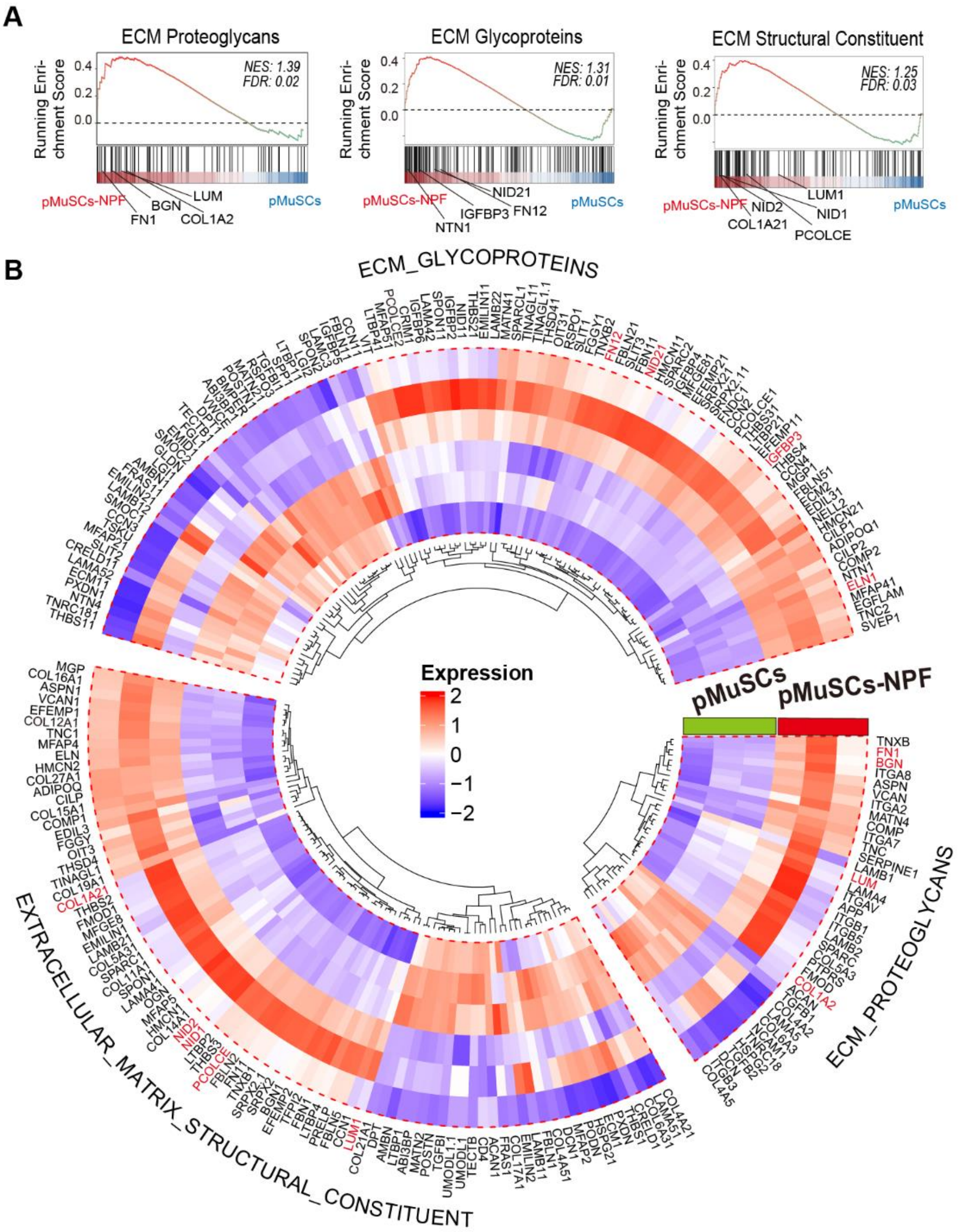

Fig. S6. The DEGs associated with ECM components in pMuSCs-NPF compared to pMuSCs. A) GSEA plots demonstrating significant enrichment of ECM proteoglycans, ECM glycoproteins, and ECM structural constituents in pMuSCs-NPF comparing to pMuSCs. NES = normalized enrichment score; FDR = false discovery rate. B) Circular heatmap of DEGs in GSEA associated with ECM proteoglycans, ECM glycoproteins, and ECM structural constituents. The outer ring highlights specific genes within each category.

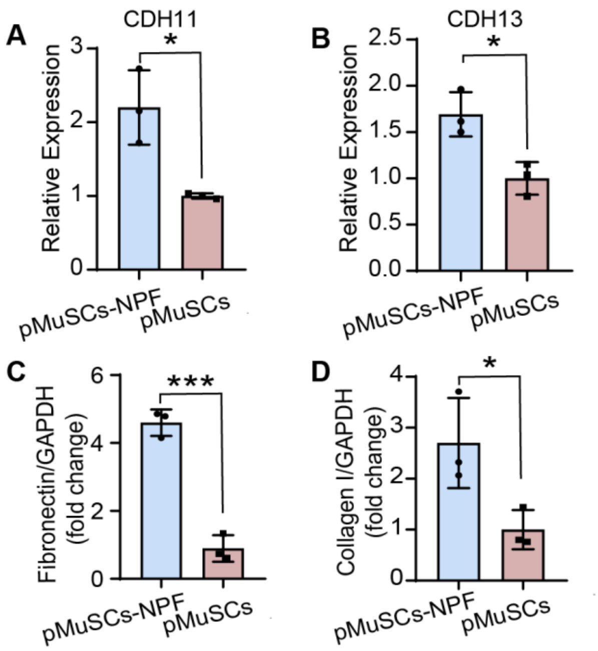

Fig. S7. Protein quantitative analysis of pMuSCs-NPF and pMuSCs. A, B) Relative expression levels of the CDH11 and CNH13 protein in pMuSCs-NPF compared to pMuSCs, as determined by proteomic profiling (n = 3). C, D) Quantification analysis of fibronectin and collagen I protein expression levels in pMuSCs-NPF and pMuSCs groups (n = 3). Results are presented as mean ± SD, statistical comparison was performed by a two-tailed t test. * $p < 0.05$, *** $p < 0.001$.

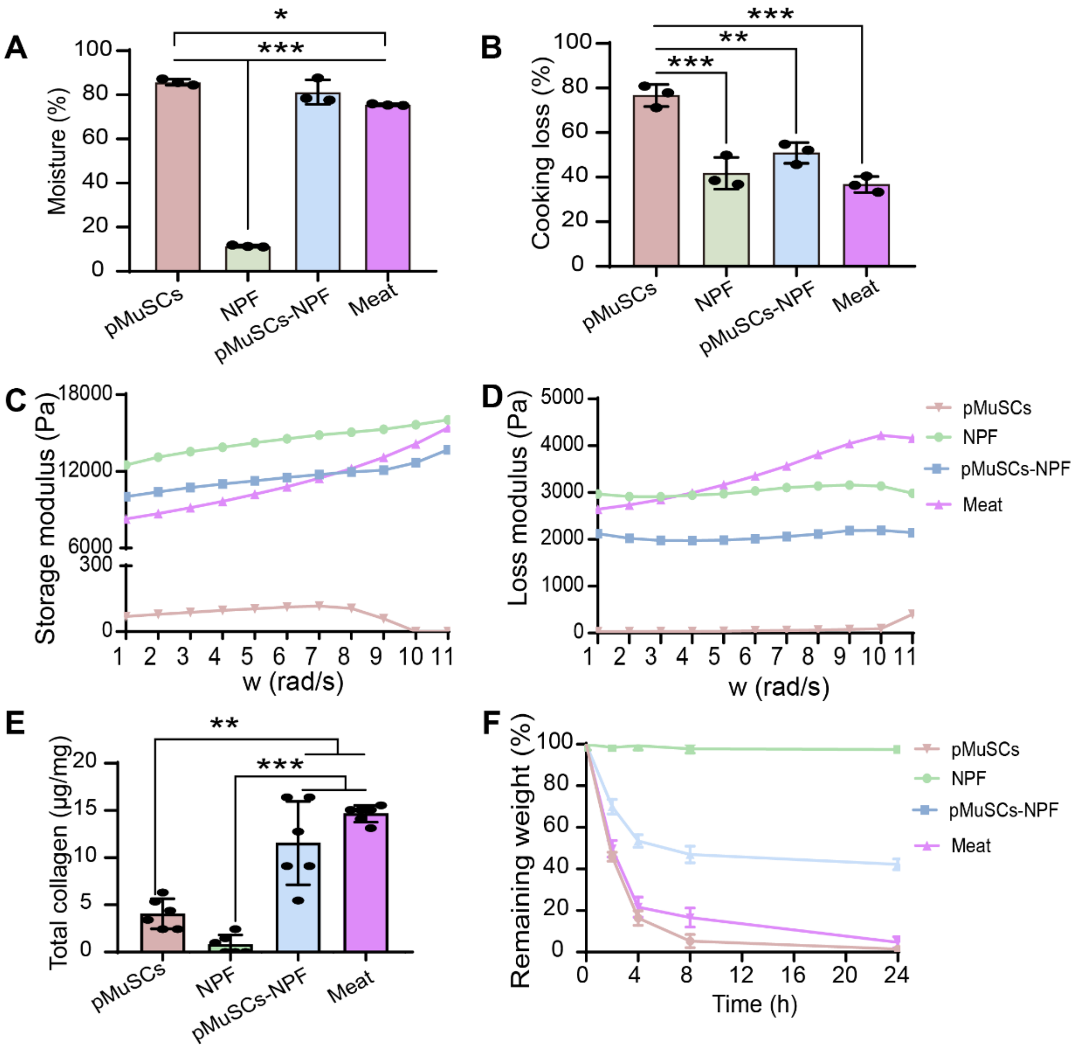

Fig. S8. Sensory characterization of the NPF scaffold-based cultured meat. A, B) Moisture content and cooking loss assessment of pMuSCs, NPF, pMuSCs-NPF, and meat (n = 3). C, D) The storage modulus (G′) and loss modulus (G″) of pMuSCs, NPF, pMuSCs-NPF, and meat (n = 3). E) Total collagen content of pMuSCs, NPF, pMuSCs-NPF, and meat (n = 6). F) *In vitro* digestion curve of pMuSCs, NPF, pMuSCs-NPF, and meat with simulated gastric fluid over time (n = 3). Results are presented as mean ± SD, One-way ANOVA with Tukey's multiple comparison tests was used. * $p < 0.05$, ** $p < 0.01$, *** $p < 0.001$.

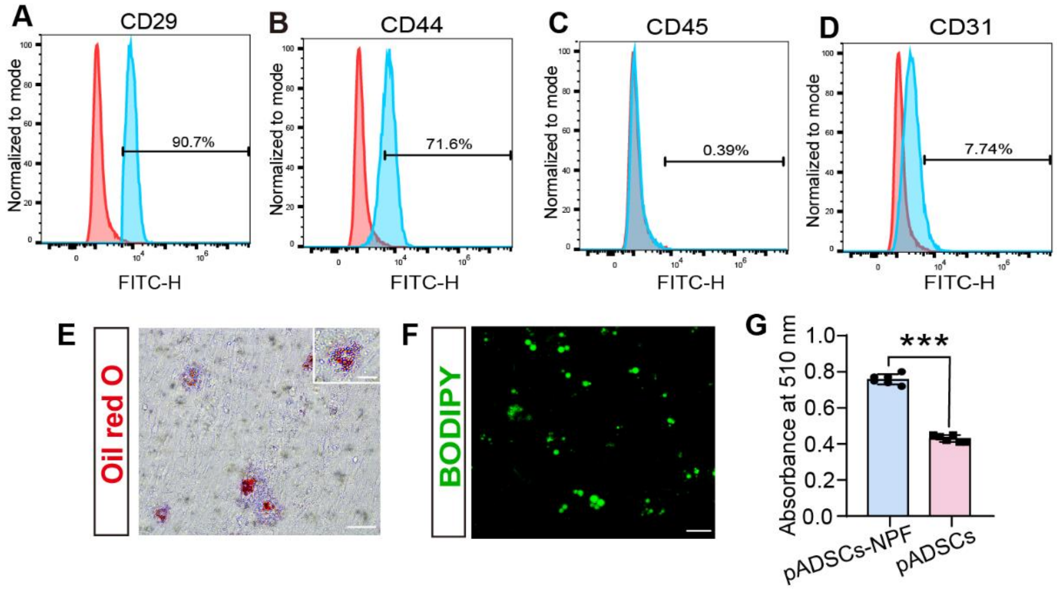

Fig. S9. Identification and adipogenesis of pADSCs. A-D) Flow cytometry analysis of the expression of cell surface markers including CD29, CD44, CD45, and CD31 in pADSC. E, F) Oil Red O (E) and BODIPY (F) immunofluorescence staining of lipid droplets in pADSCs cultured on the plate. Scale bars = 50 μm and 20 μm (insert). G) Quantitative analysis of Oil Red O staining intensity in pADSCs cultured on the NPF scaffold (pADSCs-NPF) and plate (pADSCs) (n = 6). Results are presented as mean ± SD, statistical comparison was performed by a two-tailed t test. *** $p < 0.001$.

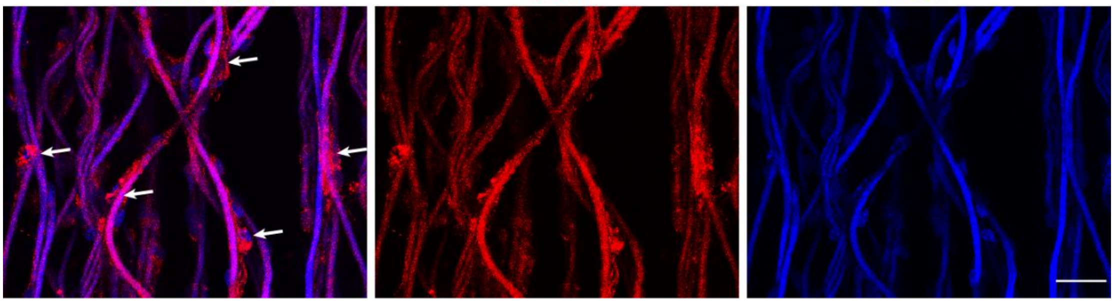

Fig. S10. Angiogenic differentiation of human umbilical vein endothelial cells (HUVECs) on the NPF scaffolds. The white arrow indicates CD31-positive cells. Scale bars = 50 μm.

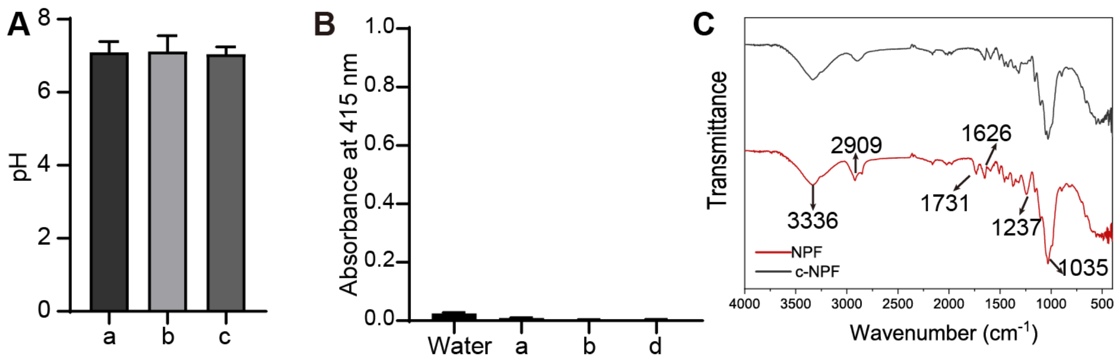

Fig. S11. Analysis of residual NaOH and H_2O_2 in scaffolds and FTIR characterization post-chemical extraction. A) pH measurements of: (a) final wash solution from NaOH/H_2O_2-treated scaffolds, (b) 24-hour soaking solution, and (c) untreated scaffold control (n=3). B) H_2O_2 quantification in: (a) final wash solution, (b) 24-hour soaking solution, (d) 24-hour simulated gastric fluid, with water as negative control (n=3). C) FTIR spectra comparing NPF and chemical extraction method's NPF (c-NPF).

Table S1. Primers for RT-qPCR.

**Primer**	**Forward**	**Reverse**
GAPDH	TCGGAGTGAACGGATTTGGC	TGACAAGCTTCCCGTTCTCC
MYOD	GCTCCGCGACGTAGATTTGA	GGAGTCGAAACACGGGTCAT
MYOG	TGACGGGGAAAACTACCTGC	ATTCACTGGGCACCCCTG
MYH2	AGGACCAAGTACGAGACGGA	AGCTTCCACGTGTTCCTCAG
MYH4	TTCTCAGGCTGCAGGACTTG	TTGGATTGTTCCTCCGCCTC
MYH7	CCGTGCTCCGTCTTCTTTCC	CGCTCCTTCTCTGACTTGCG
MYMK	CACGTTCTTCGTGGCGTTCT	ACGAAAGTCGACCTCTTGGG
DES	CCGAGATCTACGAGGAGGAG	GCTGAAAGAAGAAGCCGAGA
ACTA2	TCCAGAGCAATCAGGGGATG	AGTTGGTGATGATGCCGTGT
ITGA7	AGACGGCTTCCCAGACATTG	AATGGTTCCCATCCACGTCC

Table S2. Scoring system for meat properties.

Point	Gross appearance	Nutritional value	Texture	Juiciness	Indicated flavor	Digestibility
4	Evenly distributed light red color, shiny surface, orderly arranged fibers	Protein and collagen content close to meat (>=90%)	Hardness, springiness, chewiness, and gumminess are close to meat (>=90%)	Moisture and Cooking loss close to meat (>=90%)	Aroma, umami, sweetness, sourness, and bitterness associated amino acid close to meat (>=90%)	digestion process close to meat (>=90%)
3	Slightly unevenly distributed light red color, a little shiny surface, some fibers are arranged disorderly	70%-90% close to meat	70%-90% close to meat	70%-90% close to meat	70%-90% close to meat	70%-90% close to meat
2	Unevenly distributed red color, basically dull surface, fibers are arranged disorderly	50%-70% close to meat	50%-70% close to meat	50%-70% close to meat	50%-70% close to meat	50%-70% close to meat
1	Other colors, dull surface, fibers are arranged disorderly	Less than 50% close to meat	Less than 50% close to meat	Less than 50% close to meat	Less than 50% close to meat	Less than 50% close to meat
